# Genome-wide identification and characterization of PdbHLH transcription factors related to anthocyanin biosynthesis in colored-leaf poplar (*Populus deltoids*)

**DOI:** 10.1186/s12864-022-08460-5

**Published:** 2022-03-28

**Authors:** Xiao-jing Wang, Xu-qian Peng, Xiao-chun Shu, Yu-hang Li, Zhong Wang, Wei-bing Zhuang

**Affiliations:** 1 Key laboratory of Plant Resource Conservation and Germplasm Innovation in Mountainous Region (Ministry of Education), Guizhou University, Guiyang, 550025 Guizhou China; 2 Institute of Horticulture, Guizhou Academy of Agricultural Sciences, Guiyang, 550006 Guizhou China; 3 The Jiangsu Provincial Platform for Conservation and Utilization of Agricultural Germplasm, Institute of Botany, Jiangsu Province and Chinese Academy of Sciences (Nanjing Botanical Garden Mem. Sun Yat-Sen), Nanjing, 210014 China

**Keywords:** Colored-leaf poplar, Anthocyanin biosynthesis, *PdeHLH* genes, Phylogenetic analysis, Structure analysis, Expression pattern

## Abstract

Basic helix-loop-helix (bHLH) proteins are transcription factors (TFs) that have been shown to regulate anthocyanin biosynthesis in many plant species. However, the *bHLH* gene family in *Populus deltoids* has not yet been reported. In this study, 185 *PdbHLH* genes were identified in the *Populus deltoids* genome and were classified into 15 groups based on their sequence similarity and phylogenetic relationships. Analysis of the gene structure, chromosome location and conserved motif of the *PdbHLH* genes were performed by bioinformatic methods. Gene duplication analyses revealed that 114 *PdbHLH* were expanded and retained after WGD/segmental and proximal duplication. Investigation of cis-regulatory elements of *PdbHLH* genes indicated that many *PdbHLH* genes are involved in the regulation of anthocyanin biosynthesis. The expression patterns of *PdbHLHs* were obtained from previous data in two colored-leaf poplar (QHP and JHP) and green leaf poplar (L2025). Further analysis revealed that 12 candidate genes, including 3 genes (*PdbHLH57*, *PdbHLH143*, and *PdbHLH173*) from the subgroup III(f) and 9 gene from other groups, were positively associated with anthocyanin biosynthesis. In addition, 4 genes (*PdbHLH4*, *PdbHLH1*, *PdbHLH18*, and *PdbHLH164*) may be involved in negatively regulating the anthocyanin biosynthesis. These results provide a basis for the functional characterization of *bHLH* genes and investigations on the molecular mechanisms of anthocyanin biosynthesis in colored-leaf poplar.

**Supplementary Information:**

The online version contains supplementary material available at 10.1186/s12864-022-08460-5.

## Introduction

Colored-leaf plants played important roles in landscaping and urban beautification, which can form a nice scenery [1, 2]. Poplar is widely planted around the world due to its fast growth and better resistance to adversity, which can be used for producing timber, pulp, and paper [3]. Recently, many kinds of colored-leaf poplars have been bred, such as ‘Zhonghong polar’(ZHP), ‘Quanhong poplar’ (QHP), ‘Jinhong poplar’ (JHP) and ‘Caihong poplar’ (CHP), which also bring great economic, social and ecological benefits [4, 5]. However, the molecular mechanisms of pigment formation in colored-leaf poplar are still unclear, and needed to be exploring.

The molecular mechanisms of anthocyanins biosynthesis in many species have been well characterized, such as *Arabidopsis* and rice. The anthocyanin biosynthesis was regulated mainly by two kinds of genes, enzyme-coding structural genes and transcription factor genes [6, 7]. The structural genes are conserved in many kinds of species, mainly including phenylalanine ammonia-lyase (*PAL*), chalcone synthase (*CHS*), chalcone isomerase (*CHI*), flavanone3-hydroxylase (*F3H*), dihydroflavonol 4-reductase (*DFR*), anthocyanidin synthase (*ANS*), and flavonoid 3-O-glucosyltransferase (*UF3GT*), and the stable anthocyanins can be synthesized by various modifications, such as methylation, glycosylation and acylation [8, 9]. Several transcription factors have been reported to be involved in the anthocyanin biosynthesis, mainly including MYB, bHLH and WD40. The MYB transcription factors can regulate the anthocyanin biosynthesis independently or form MYB-bHLH-WD40 (MBW) complexes to regulate the anthocyanin biosynthesis [6, 10]. Up to now, many MYB TFs associated with anthocyanins biosynthesis has been studied in poplar, such as PtrMYB116, PtrMYB117, PtrMYB118, PtrMYB119, PtrMYB120 [11–13]. However, the functions of bHLH TFs in poplar have been investigated fewer compared with these of MYB TFs.

The bHLH TFs are the second largest family of plant TFs, and play a central role in anthocyanins biosynthesis [14, 15]. The conserved bHLH domain contains 50 to 60 amino acids and processes two functional regions: basic region and HLH region [16]. The basic region contained approximately 17 amino acids, which located at the N-terminus of bHLH domain and binded to a consensus hexanucleotide E-box (CANNTG) [17]. The HLH region with 50 amino acids was used to form the homodimers or heterodimers, which includes two alpha helixes separated by a variable loop [18]. bHLH TFs are involved in several plant metabolic pathways including flavonoids and anthocyanin biosynthesis [19]. The Lc protein was the first reported bHLH TFs in maize, which can be involved in the anthocyanin biosynthesis through regulating at least 2 structural genes [20]. Moreover, several bHLH TFs related to anthocyanin biosynthesis were identified and further characterized in other plants, such as *Arabidopsis* (*AtEGL3*, *AtGL3*, and *AtTT8*) [21], *V. vinifera* (*VvMYCA1*) [22], *N. tabacum* (*NtAn1* and *NtAn2*) [23], *M. domestica* (*MdbHLH3* and *MdMYC2*) [24, 25] and poplar (*PdTT8*) [26]. In poplar, PdTT8 directly interacts with PdMYB118 TF to regulate wound-induced anthocyanin biosynthesis [26]. However, several questions are still needed to be explored. Is there other bHLH TFs involved in the anthocyanin biosynthesis in poplar? What’s the difference among the different bHLH TFs associated with anthocyanin biosynthesis in different species? What’s the difference among the different bHLH TFs involving the anthocyanin biosynthesis in poplar? The genome-wide identification and characterization of *bHLH* gene family in poplar could give us the answer of these questions.

The genome-wide identification and characterization of *bHLH* gene family has been conducted in many plants [14, 27, 28]. There is 162 *bHLH* genes in *Arabidopsis*, 192 in tobacco, 159 in tomato, and 188 in apple, which can be divided into 15–26 subfamilies [28]. Among these subfamilies, members of the III subfamily have been proved to be involved in anthocyanin synthesis [29]. Therefore, bHLH TF in poplar falling into the III subfamily might also be involved in anthocyanin synthesis. In present study, the phylogenetic analysis, gene or protein structures, gene duplication, and chromosome distribution of PdbHLH transcription factors were systematically and comprehensively investigated. Moreover, the expression pattern of *PdbHLH* genes in green leaf poplar (L2025) and colored leaf poplar (QHP) was evaluated with the released RNA-seq data [5]. To better explore the functions of *PdbHLH* genes in poplar, the expression pattern of *PdbHLH* genes in green leaf poplar (L2025) and colored leaf poplar (JHP) was further analyzed with the released RNA-seq data [30]. Our findings should not only provide a characterization of the *PdbHLH* gene superfamily but also provide insight into the roles of *PdbHLH* genes in the regulation of anthocyanin biosynthesis in colored-leaf poplar.

## Methods

### Identification of *PdbHLH* gene family in *P. deltoids*

The genome sequence and corresponding annotations of *P. deltoids* was downloaded from the DOE Joint Genome Institute website [31] (http://genome.jgi.doe.gov/). The hidden Markov Model profile of HLH (PF00010) domain obtained from Pfam database [32] (http://pfam.xfam.org/) was used to search candidate *PdbHLH* genes from *P. deltoids* genome using the HMMER3 software package [33]. Moreover, the physical localizations of all candidate genes and redundant sequences with the same chromosome location and short proteins (length < 100 aa) were further checked. Next, the Pfam [32] and SMART [34] (http://smart.embl-heidelberg.de/) databases was used to further verify the presence of HLH domain for all candidate protein sequences. After the above three steps, the identified protein sequences that contained the core domain (HLH) of known PdbHLH were regarded as putative homologs in the study.

### Sequence analysis and structural characterization of *PdbHLH* genes in *P. deltoids*

Based on the genome sequence and annotation information, the exon-intron organization of *PdbHLH* genes, including intron distribution, number, and phases, was graphically displayed by the Gene Structure Display Server GSDS2.0 (http://gsds.cbi.pku.edu.cn/). The MEME suite (http://meme-suite.org/tools/meme) was used to identify the conserved motifs of PdbHLH proteins [35]. The optimized parameters were employed for the analysis as follows: maximum numbers of different motifs, 20; minimum motif width, 6 bp; maximum motif width, 50 bp.

### Chromosome distribution, gene duplication, and synteny analysis of *PdbHLH* genes in *P. deltoids*

The chromosome distribution of *PdbHLH* genes was extracted from the genome annotation database, and the MapChart software was used to visualize the chromosomal locations of the *PdbHLH* gene [36]. Gene duplication analyses for *P. deltoids* was conducted using the Multiple Collinearity Scan Toolkit (MCScanX) [37]. To identify candidate homologous gene pairs (E < 1e^− 5^), BLASTp was performed to search for potential homologous gene pairs across the whole *P. deltoids* genome. The potential homologous gene pairs were inputted into the program MCScanX with the default parameters to identify syntenic chains. MCScanX was used to further distinguish among whole-genome duplication (WGD)/segmental, dispersed proximal, and tandem duplication events in *PdbHLH* gene family [37]. Gene pairs identified in the same synteny block were used to calculated Ka and Ks values using PAML package [38].

### Cis-elements in the promoter regions of *PdbHLH* genes and GO enrichment analyses

Conserved cis-regulatory elements in the promoter region of *PdbHLH* genes were identified by analyzing the 2000-bp sequence upstream of the transcription start site (TSS) obtained from TBtools. Promoter sequence analysis was performed using PlantCARE [39, 40].

GOATOOLS (http://github.com/tanghaibao/GOatools) was used to perform GO annotations for *PdbHLH* genes. The biological function enrichment analysis for *PdbHLH* genes was conducted using Fisher’s exact test. Moreover, the Bonferroni multiple testing correction was used to minimize false positives, and functions were considered to be significantly enriched when their Bonferroni-corrected *P*-values (Padjust) were < 0.05.

### Phylogenetic relationships analysis of *PdbHLH* gene family in *P. deltoids*

The bHLH protein sequences, including PdbHLH, OsbHLH, and AtbHLH proteins from poplar, rice and Arabidopsis were aligned using the Muscle algorithm with default parameters. The best-fit model of protein evolution was selected using the Model-Generator program [41]. An unrooted neighbor-joining phylogenetic tree was constructed through multiple sequence alignments of these bHLH proteins using MEGA 10.0, and these bHLH proteins were further grouped into different clades based on the topology of the phylogenetic tree. The parameters were as follows: pairwise deletion, Poisson model, and 1000 bootstrap replications.

### Expression analyses of *PdbHLH* genes in ‘Jinghong poplar’ (JHP), ‘Quanhong poplar’ (QHP) and *Populus* sp. Linn. 2025 (L2025) by RNA-seq

RNA-seq data for the *PdbHLH* genes were obtained from previous studies of differential gene expression in colored-leaf polar (JHP and QHP) and green leaf poplar L2025 [5, 30] RNA-seq data for each *PdbHLH* were extracted, analyzed, normalized, and displayed in heat maps. The transcript abundance of *PdbHLH* genes was calculated as fragments per kilobase of exon model per million mapped reads (FPKM). The log_2_ (FPKM) from the RNA-seq data were subjected to hierarchical clustering with Cluster 3.0, and the results were graphically displayed using Java TreeView [35].

### Validation of RNA-seq reliability in the leaves of JHP, QHP and L2025

To validate the reliability of RNA-seq results, the expression levels of 9 *PdbHLH* genes associated with anthocyanin biosynthesis in the leaves of JHP, QHP and L2025 were evaluated by qRT-PCR using an Applied Biosystems 7500 Real-Time PCR system (Applied Biosystems, Waltham, MA, USA). Gene-specific primers were designed according to the sequence of *PdbHLH* genes (Table S1), and the *ACTIN2* gene was used as a control gene [12]. The relative expression levels of 9 genes were analyzed using SPSS 17.0 with three biological replicates.

### Protein interaction prediction

To investigate potential differential proteins that interact with PdbHLH proteins involved in anthocyanin biosynthesis, the putative PdbHLH protein sequences associated with anthocyanin biosynthesis were screened according to their phylogenetic analysis and expression pattern, and submitted to the online server STRING v10 (https://string-db.org). The PPI networks were constructed based on the active interaction sources with required confidence score > 0.4 in *Arabidopsis*, which included biological experiments, co-expression and databases. The PPI networks were visualized by Cytoscape software (v. 3.8.1). ClusterONE software was used to detect highly connected regions of the network, and the criteria are as follows: minimum density = 0.01, minimum size = 2 and edge weights = combined_score. The nodes and edges in the networks represent proteins and interactions, respectively. The interactions between bHLH proteins involved in anthocyanin biosynthesis and potential differential proteins in JHP and QHP were screened by STRING.

## Results

### Identification and chromosome distribution of the *PdbHLH* gene family in *P. deltoids*

**Fig. 1:**
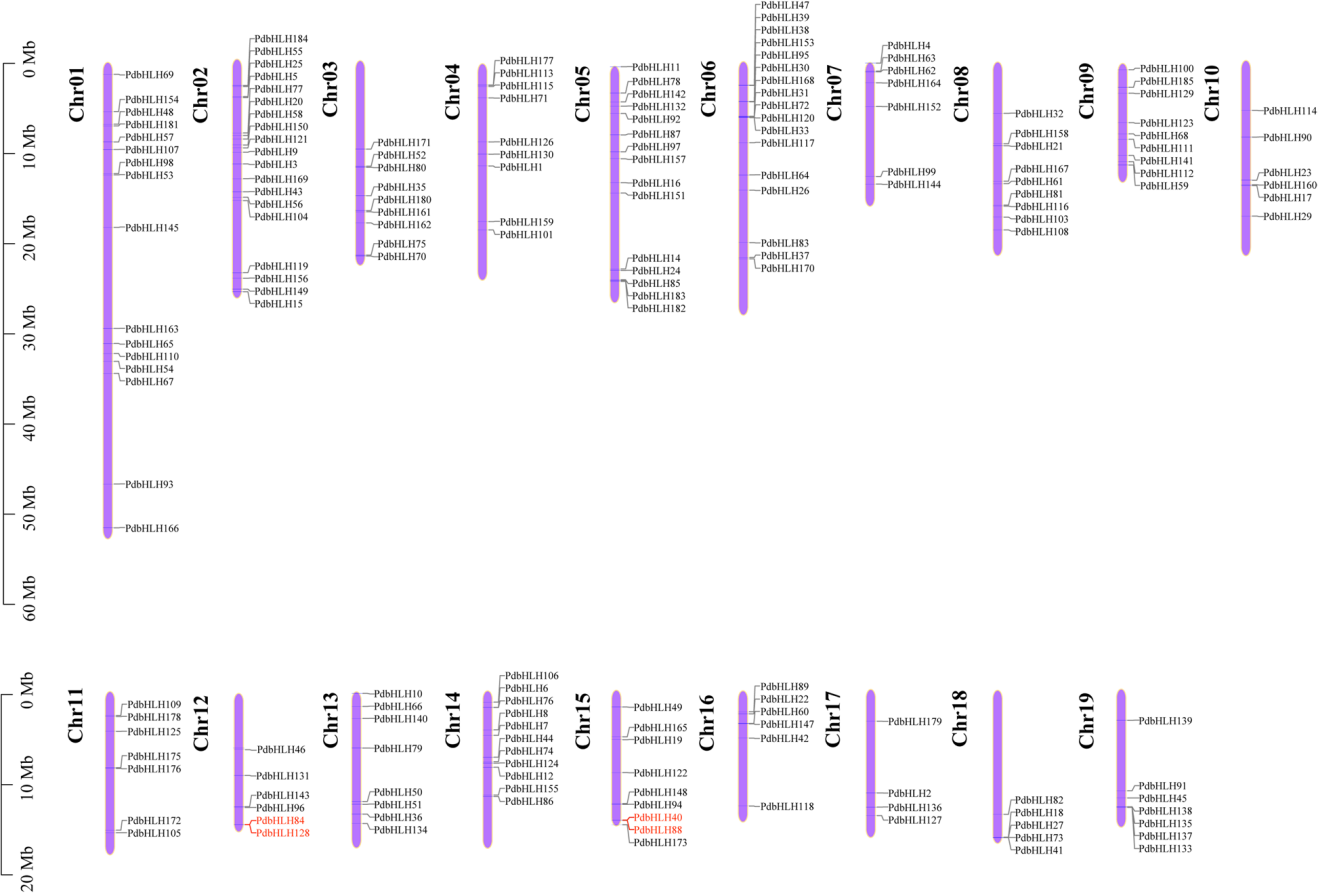
Chromosome distribution and tandem duplication events for *PdbHLH* genes. The position of each *PdbHLH* is noted on the right side of each chromosome (Chr). The size of a chromosome is indicated by its relative length. Tandemly duplicated genes are indicated with a red bar

There are 185 PdbHLH family proteins identified in *P. deltoids (*Table S2). The PdbHLH proteins range in size from 129 (PdbHLH72) to 943 (PdbHLH185) amino acids, with an average length of approximately 373 amino acids. The molecular weight of the identified PdbHLH proteins ranged from 14.95 kDa (PdbHLH72) to 103.49 kDa (PdbHLH185), and the predicted isoelectric points of these proteins ranged from 4.62 (PdbHLH58) to 9.46 (PdbHLH62). In addition, the subcellular localization of the identified PdbHLH proteins was also predicted, and 181 of 185 (approximately 97.8%) PdbHLH proteins were localized in the nucleus (Table S2). According to the information of gene annotation, the predicted 185 *PdbHLH* genes were localized on poplar chromosomes. As shown in Fig. 1, the congregate region and the number of *PdbHLH*s is unevenly although the *PdbHLH* genes distributed on all of the 19 chromosomes. Chromosome 2 possessed the largest number *PdbHLH* genes (19), while only four *PdbHLH* genes were present on chromosome 17. The percentage of *PdbHLH* genes per chromosome varied from 0.23% on chromosome 17 to 0.65% on chromosome 2 (Table S3).

### Phylogenetic analysis of the *PdbHLH* gene family among *Arabidopsis,* rice, and *P. deltoids*

**Fig. 2:**
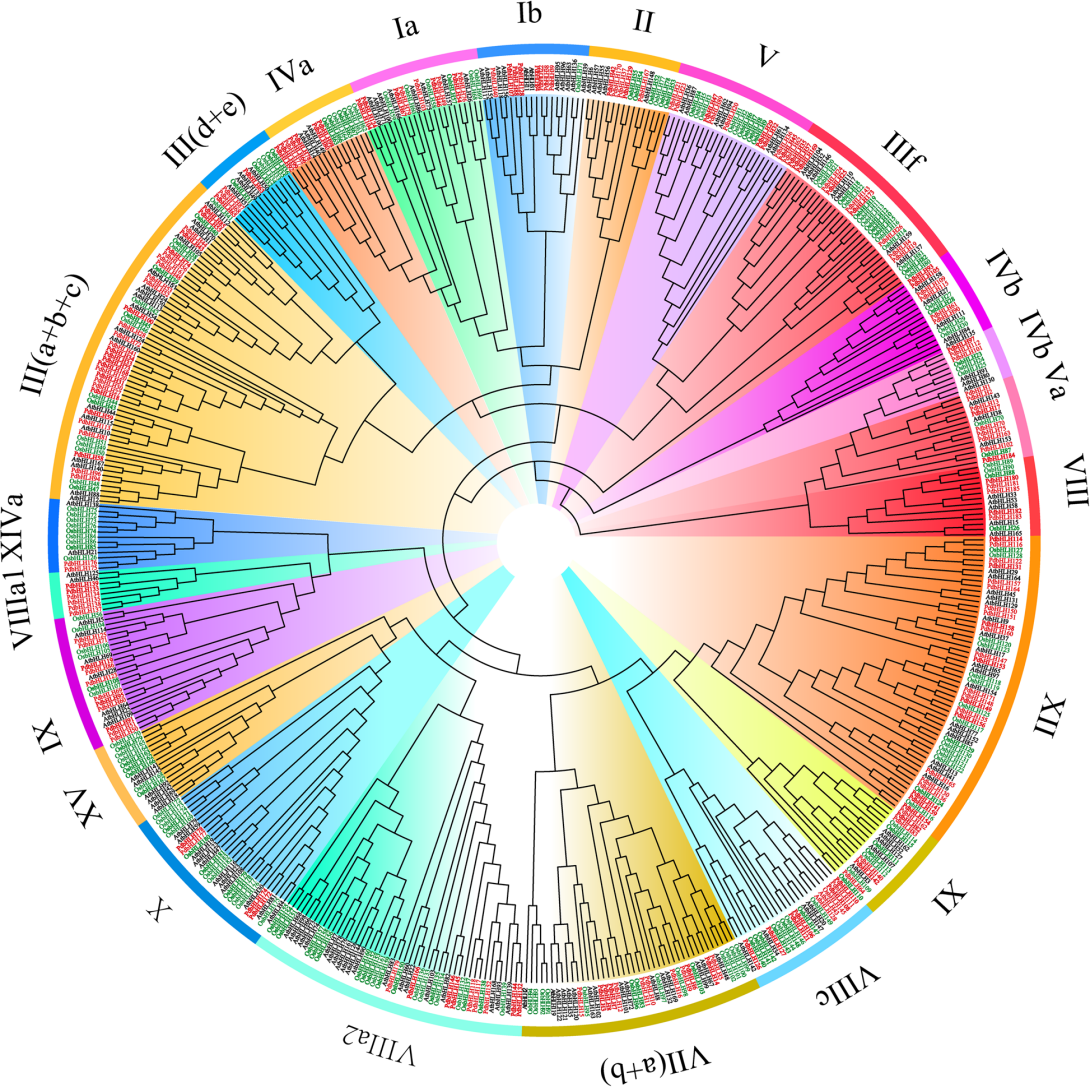
Phylogenetic analysis of *bHLH* gene family in *Arabidopsis*, rice and *P. deltoids.* An un-rooted phylogenetic tree of *bHLH* gene family among *Arabidopsis*, rice and *P. deltoids* was constructed using the neighbor-joining method in MEGA 10.0 software with a bootstrap test (replicated 1000 times). The *bHLH* gene families in *Arabidopsis*, rice and *P. deltoids* were marked black, dark green and red, respectively

An unrooted phylogenetic tree of the bHLH family genes in *P. deltoids Arabidopsis*, and rice was constructed using the neighbor-joining method with the default parameters of MEGA 10.0 (Fig. 2). The PdbHLH family members of *P. deltoids* were divided into 15 groups (I to XV) according to the topology of the phylogenetic tree, as well as the classification and nomenclature of bHLH proteins in *Arabidopsis* and rice. The number of *PdbHLH* genes in different group was different. Group III, containing 37 *PdbHLH* genes, is the largest group in the 15 groups, which was further divided into 5 subgroups (subgroup a-e). Group XII and V contained 24 and 19 *PdbHLH* genes, respectively. Group XV only contained the *bHLH* genes of *Aranidopsis* and rice, and didn’t include the *bHLH* gene of *P. deltoids*. Apart from the groups II, IV, V, and XII, the other groups contained much more *bHLH* genes in *P. deltoids* than those in *Arabidopsis* and rice. In addition, some groups were further classified into two or more subgroups (Fig. 2). Subgroup VIII_a1_, and IIIa-c mainly contained the *bHLH* gene clusters in *P. deltoids*, indicating that the species-specific expansion of these genes occurred in *P. deltoids* after the divergence of core eudicots. In addition, some subgroups just contained *AtbHLH* or *OsbHLH* genes without *PdbHLH* genes, suggesting that *bHLH* genes could have been either acquired and expanded in *Arabidopsis* or rice during evolution process or specially lost in *P. deltoids*.

### Gene structure and protein motif analysis of the *PdbHLH* gene family in *P. deltoids*

**Fig. 3:**
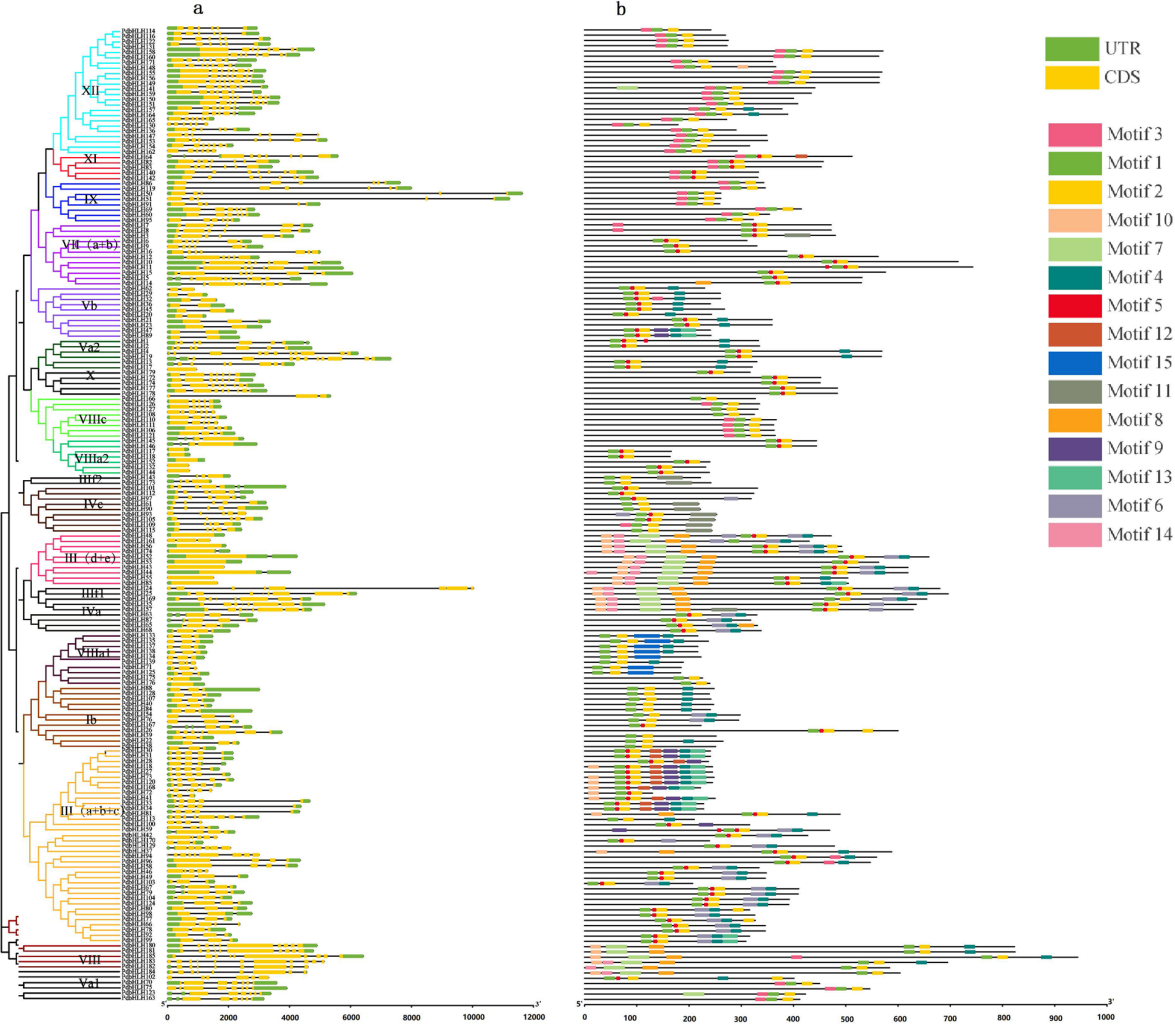
Schematic diagram of exon/intron distribution and amino acid motifs of *PdbHLH* family genes in *P. deltoids*. **a** Exon/intron distribution of of *PdbHLH* genes in *P. deltoids*. The exons are represented by orange round-cornered rectangles. The black lines connecting two exons represent introns. **b** Distribution of conserved motifs in each PdbHLH proteins. Schematic diagram of motif structure in *P. deltoids* PdbHLH gene family using MEME. The relative positions of each conserved motif within the PdbHLH proteins are shown in color. The black lines represent the non-conserved sequences

To better analyze the structural diversity and motif composition of *PdbHLH* genes, the investigation of intron and exon distribution profile was conducted and visualized using the Gene Structure Display Server 2.0 (GSDS, Fig. 3). A total of 185 *PdbHLH* genes possessed exons varying from 1 to 12. Fourteen *PdbHLH* genes lacked introns and had only one exon, including *PdbHLH43*, *PdbHLH48*, *PdbHLH53*, *PdbHLH54*, *PdbHLH55*, *PdbHLH56*, *PdbHLH85*, *PdbHLH117*, *PdbHLH118*, *PdbHLH132*, *PdbHLH144*, *PdbHLH152*, *PdbHLH176*, *PdbHLH179*. The majority (171 of 185) of the *PdbHLH* genes have 2 to 8 introns, and *PdbHLH19* contained 12 exons and 11 introns, which was the greatest number of exons in the total *PdbHLH* genes. As expected, gene structure analysis showed that most members in the same group had similar intron/exon compositions, including the numbers and length of exons. For example, all the members of subfamily XV have more than 5 exons, and all the members of group X have 3 exons. Thus, our results indicate that *PdbHLH* genes in the same group or subgroup had similar gene structures, further verify the phylogenetic relationship of these *PdbHLH* genes (Fig. 2).

There were 15 putative conserved protein motifs identified in the PdbHLH proteins through MEME analysis (Motifs 1–15, Fig. 3, Fig. S1). Motifs 1 the basic region and the first helix, and Motif 2 contained the second helix, both of which correspond to the bHLH domain. The bHLH domain has been identified in all PdbHLH proteins. Seven *PdbHLH* genes, including *PdbHLH38*, *PdbHLH39*, *PdbHLH175*, *PdbHLH176*, *PdbHLH166*, *PdbHLH127* and *PdbHLH108*, only contained the Motifs 1 and Motif 2. In each group, the components of the conserved motifs for most of the proteins were similar (Fig. 3b). For example, Motif 11, Motif 9, and Motif 15 were specifically distributed in the groups V, X, and XII, respectively. Motifs 6-8, 10-14 were commonly identified in all members of group IX.

### Pivotal cis-elements in the promoter of *PdbHLH* genes in *P. deltoids*

Cis-regulatory elements, which are usually restricted to 5′ upstream areas of genes, are the binding sites of TFs, and are responsible for transcriptional regulation. Thus, the 2000 bp upstream of the transcription start site (TSS) of *PdbHLH* genes were used to explore gene regulation patterns with PlantCARE (Fig. S2). There were 23 functionally annotated cis-elements in the promoter of most *PdbHLH* genes, which were roughly divided into three categories: light-responsive elements (GT1-motif, I-box, AT1-motif, G-box, GATA-motif, GA-motif, Box II, Gap-box, L-box, CGTCA-motif, CAG-motif, AAAC-motif, and GTGGC-motif), stress-responsive elements [MYB-recognizing element (MRE), MYB-binding site (MBS), GC-motif, TC-rich repeats, LTR, and CCAAT-box]; and hormone-responsive elements (TCA-element, P-box, ABRE, TGACG-motif, TGA-element, GARE-motif, TATC-box, and SARE). The presence of MRE and MBS cis-elements in the *PdbHLH* gene promoter suggests that bHLH proteins might be transcriptional regulated by MYB TFs in *P. deltoids* to modulate the expression of downstream targets.

### GO enrichment analysis of the *PdbHLH* genes in *P. deltoids*

**Fig. 4:**
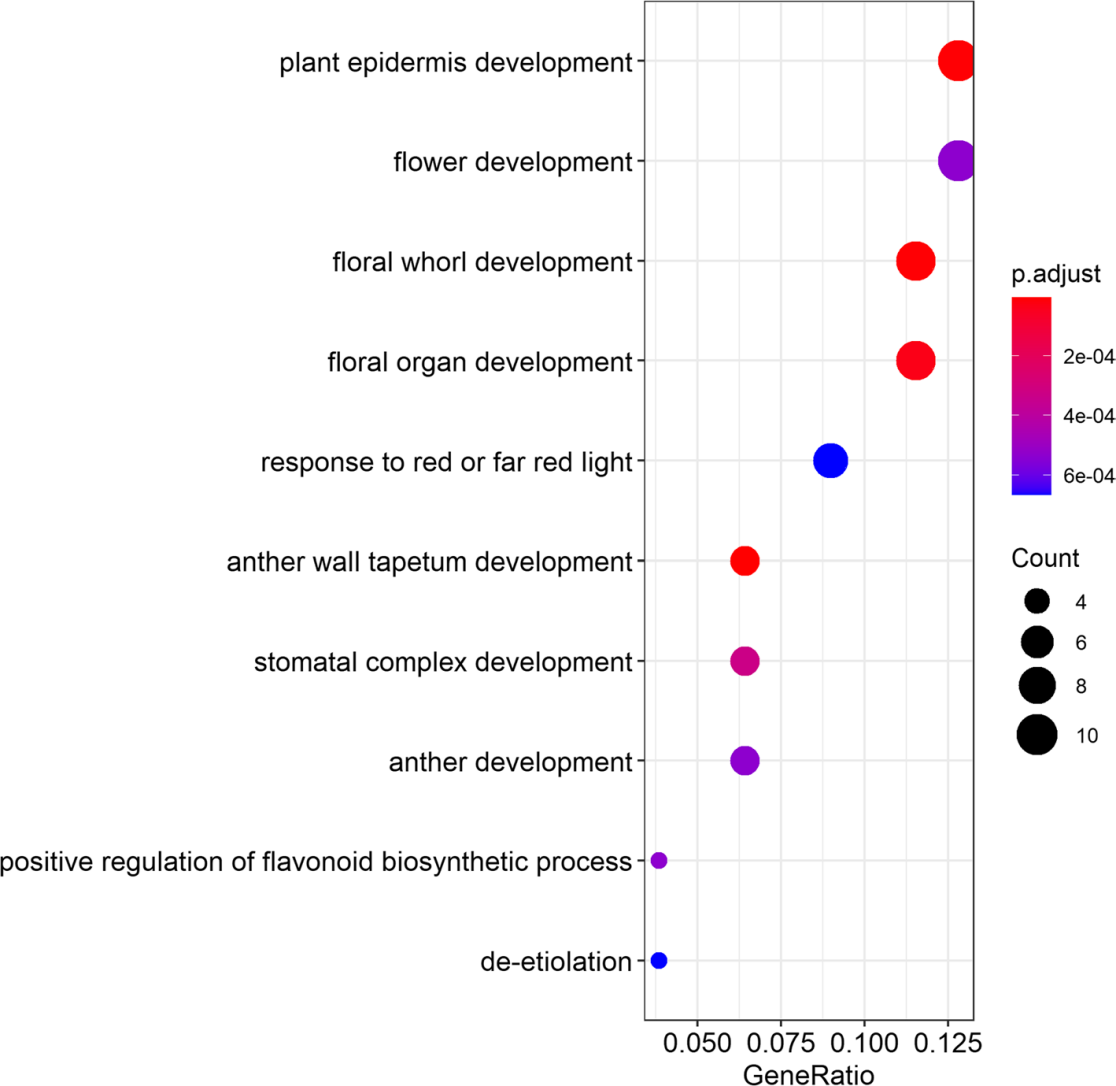
GO enrichment analysis of the PdbHLH proteins relative to the GO database. The vertical axis indicates the enrichment factor, and the size of the circle indicates the number of genes annotated with a given GO term. GOATOOLS (http://github.com/tanghaibao/GOatools) was used to assign GO annotations to PdbHLHs, and Fisher’s exact test was used to identify biological functions enriched in the PdbHLHs relative to the full GO database. Visualization was performed using the Majorbio online platform (https://cloud.majorbio.com)

The bHLH proteins play a central role in a wide range of metabolic, hysiological, and developmental processes in higher plants. To further investigate the biological functions of the *PdbHLH* genes in *P. deltoids*, gene ontology (GO) annotation and enrichment analysis of the 185 *PdbHLH* genes were performed in present study (Fig. 4 and Table S4). Six molecular functions, two cellular components, and fifty-two biological processes in GO terms were enriched in the *PdbHLH* genes relative to the complete GO database. In the biological process category, *PdbHLH* genes were mainly enriched in flower development (*n* = 10), plant epidermis development (*n* = 10), floral organ development (9), floral whorl development (9), plant organ morphogenesis (7), response to red or far red light (7) and so on. In the cellular component category, the genes were enriched in RNA polymerase II transcription (5) and transcription regulator complex (5). In the molecular function category, the genes were enriched in DNA-binding transcription activator activity (14), DNA-binding transcription activator activity and RNA polymerase II-specific (13), DNA-binding transcription factor activity and RNA polymerase II-specific (13), RNA polymerase II transcription regulatory region sequence-specific DNA binding (7), identical protein binding (7) and transcription factor binding (5). GO enrichment results suggested that PdbHLH TFs mainly involved in nucleic acid-binding TF activity, catalytic activity, developmental processes of cellular and multi-organism.

### Gene duplication events of the *PdbHLH* gene family in *P. deltoids*

Several gene duplication events, including WGD or segmental duplication, tandem duplication, dispersed gene duplication and others, promote the evolution of protein-coding gene families [42]. In the present study, the origins of duplicate genes for the *PdbHLH* gene family in *P. deltoids* genome was detected with MCScanX package. Each member of *PdbHLH* gene family was assigned to one of five different categories: singleton, WGD/segment duplication, tandem, proximal and dispersed. Remarkably, 31 (16.7%), 57 (30.8%) and 57 (30.8%) of the *PdbHLH* family genes in *P. deltoids* were duplicated and retained from singleton, proximal, and whole genome duplication (WGD)/segmental duplication, respectively (Fig. S3). Only five *PdbHLH* genes originated from tandem duplication. However, there was no *PdbHLH* genes originated from proximal in our results (Fig. S3).

**Fig. 5:**
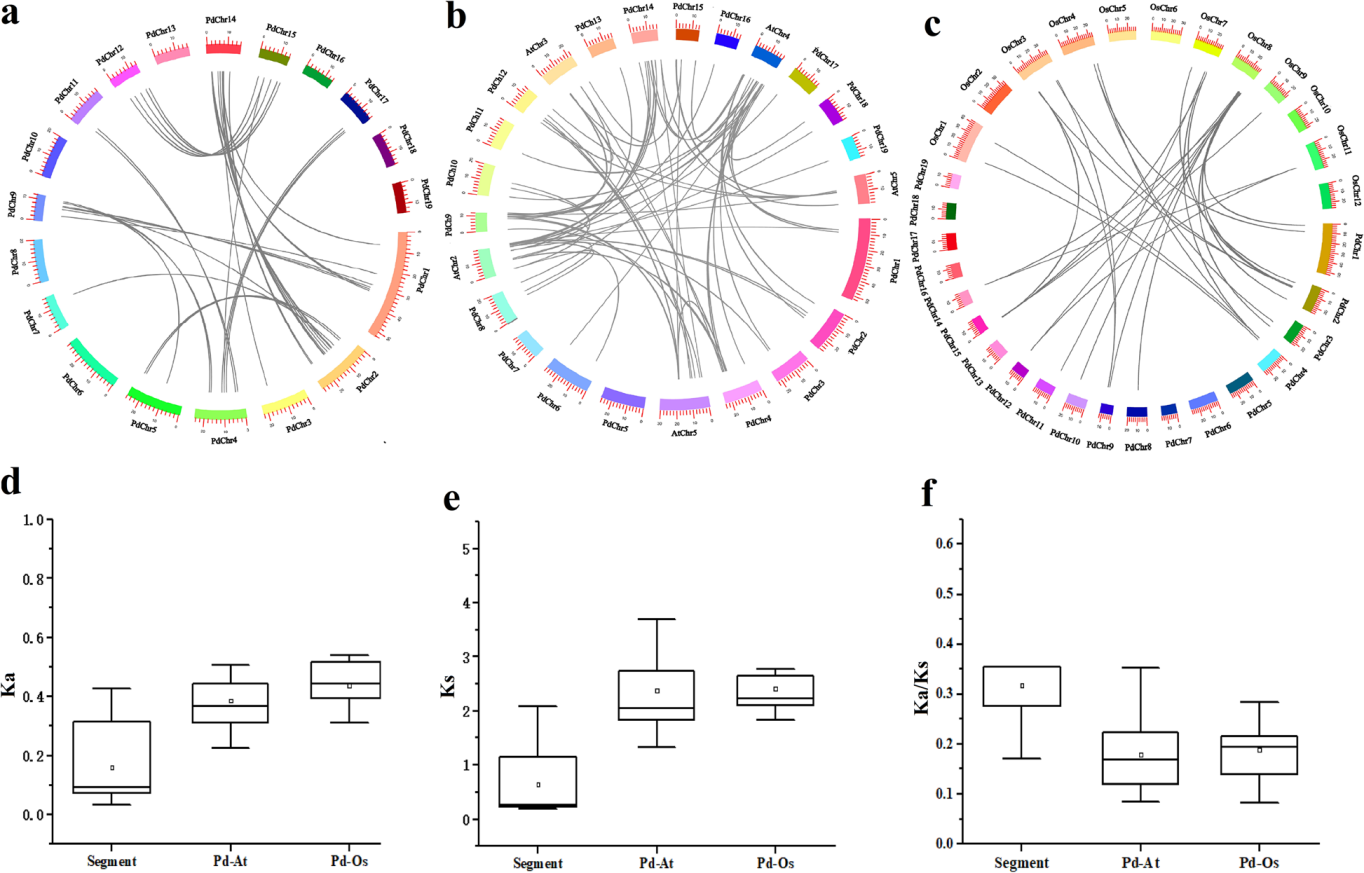
Genomic locations of *PdbHLH* genes and segmentally duplicated gene pairs in the *P. deltoids* genome (**a**) and the orthologous relationships of PdbHLH TFs with *Arabidopsis* (**b**) and rice (**c**). The black lines in a indicated 33 segmentally duplicated *PdbHLH* gene pairs. The chromosome number is indicated at the top of each chromosome. Ka (**d**), Ks (**e**), and Ka/Ks (**f**) ratio of segmental duplicate genes and orthologous genes among rice, *Arabidopsis*, and *P. deltoids*. The box plots are exhibiting the distributions of Ka, Ks, and Ka/Ks values among paralogs and orthologs. The small square and the line in the box represent average and median values of the Ka, Ks, and Ka/Ks values, respectively. Pd: *P. deltoids*; At: *Arabidopsis*; Os: Rice

To further explore the potential evolutionary mechanisms of *PdbHLH* gene family, the collinearity analysis of the *PdbHLH* gene family in *P. deltoids* genome was performed using the all-vs.-all local BLASTP and MCScan methods (Fig. 5). A total of 33 segmental duplication events with 52 *PdbHLH* genes were identified in the *P. deltoids* genome, which accounted for 91.2% of WGD-type *PdbHLH* genes (Fig. 5 and Table S5). *PdbHLH* genes were located within synteny blocks on all chromosomes. Subsequently, non-synonymous to synonymous substitution ratio (Ka/Ks) was calculated using the pairwise model by maximum likelihood (PAML v8.0, Table S5). Ka/Ks < 1 indicates purifying selection, whereas Ka/Ks > 1 is the signature of positive selection (Hurst, 2002). In the present study, the Ka/Ks ratios of 32 *PdbHLH* gene pairs were less than one, implying that these genes are under negative purifying selection, which maintained the functions of the *PdbHLH* gene family in *P. deltoids.* Moreover, Ks was usually used to estimate the evolutionary dates of genome or gene duplication events. The WGD/segmental duplicated events in *P. deltoids* occurred from 6.42 (Ks = 0.1927) to 90.14 mya (Ks = 2.8244).

The orthologous relationships of the *PdbHLH* family genes among *Arabidopsis,* Rice and *P. deltoids* were also investigated by collinearity analysis (Fig. 5, Table S6 and Table S7). There were 56 orthologous bHLH gene pairs between *P. deltoids* and *Arabidopsis* and 20 orthologs between *P. deltoids* and rice (Fig. 5). The number of orthologous events of PdbHLH-AtbHLH was much greater than that of PdbHLH-OsbHLH. The details of the collinear bHLH gene pairs were provided in Table S6 and S7.

### Expression profile of the *PdbHLH* genes in colored-leaf poplar JHP and green-leaf poplar L2025

**Fig. 6:**
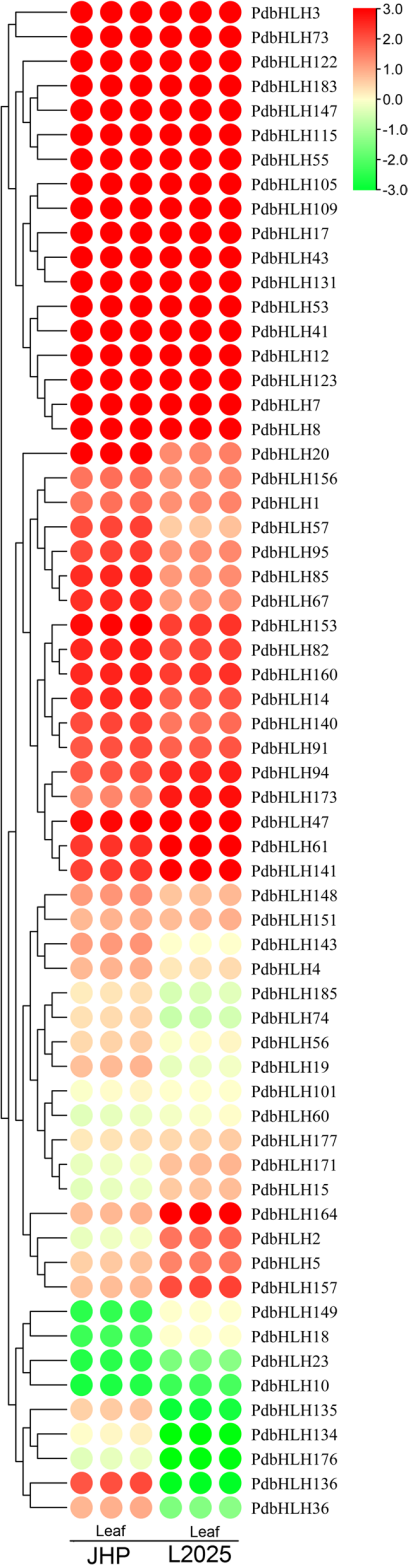
Gene expression pattern of *PdbHLHs* in the leaves of JHP and L2025 by RNA-seq. The scale bars represent the log_2_ transformations of the RPKM values

To evaluate the expression pattern of *PdbHLH* genes in colored-leaf poplar, the expression profiles of *PdbHLH* genes in the leaves of JHP and L2025 was evaluated using the previous RNA-seq data (Fig. 6, S4 and Table S8). The expression level of candidate *PdbHLH* genes in the leaves of JHP was more than 10 times that these in L2025, including 1 *PdbHLH* gene from Group I, 10 *PdbHLH* genes from Group III, 4 *PdbHLH* genes from Group IV, 4 *PdbHLH* genes from Group V, 5 *PdbHLH* genes from Group VII, 3 *PdbHLH* genes from Group IX, 2 *PdbHLH* genes from Group XI, 9 *PdbHLH* genes from Group XII, and 1 *PdbHLH* gene from Group XIII. These candidate *PdbHLH* genes are shown in Table S8. Among them, the expression levels of 12 *PdbHLH* genes (*PdbHLH141*, *PdbHLH95*, *PdbHLH140*, *PdbHLH57*, *PdbHLH136*, *PdbHLH91*, *PdbHLH94*, *PdbHLH156*, *PdbHLH1*, *PdbHLH173*, *PdbHLH148*, and *PdbHLH143*) were more than 100 times than that in L2025. In addition, the expression level of 23 *PdbHLH* genes in the leaves of L2025 was more than 10 times that these in JHP (Table S8). Among them, the expression level of 2 genes (*PdbHLH4* and *PdbHLH 36*) in the leaves of L2025 was more than 40 times that these in JHP. These results suggested that these genes might be involved in regulating the anthocyanin biosynthesis.

### Expression profile of the *PdbHLH* genes in colored-leaf poplar QHP and green-leaf poplar L2025

**Fig. 7:**
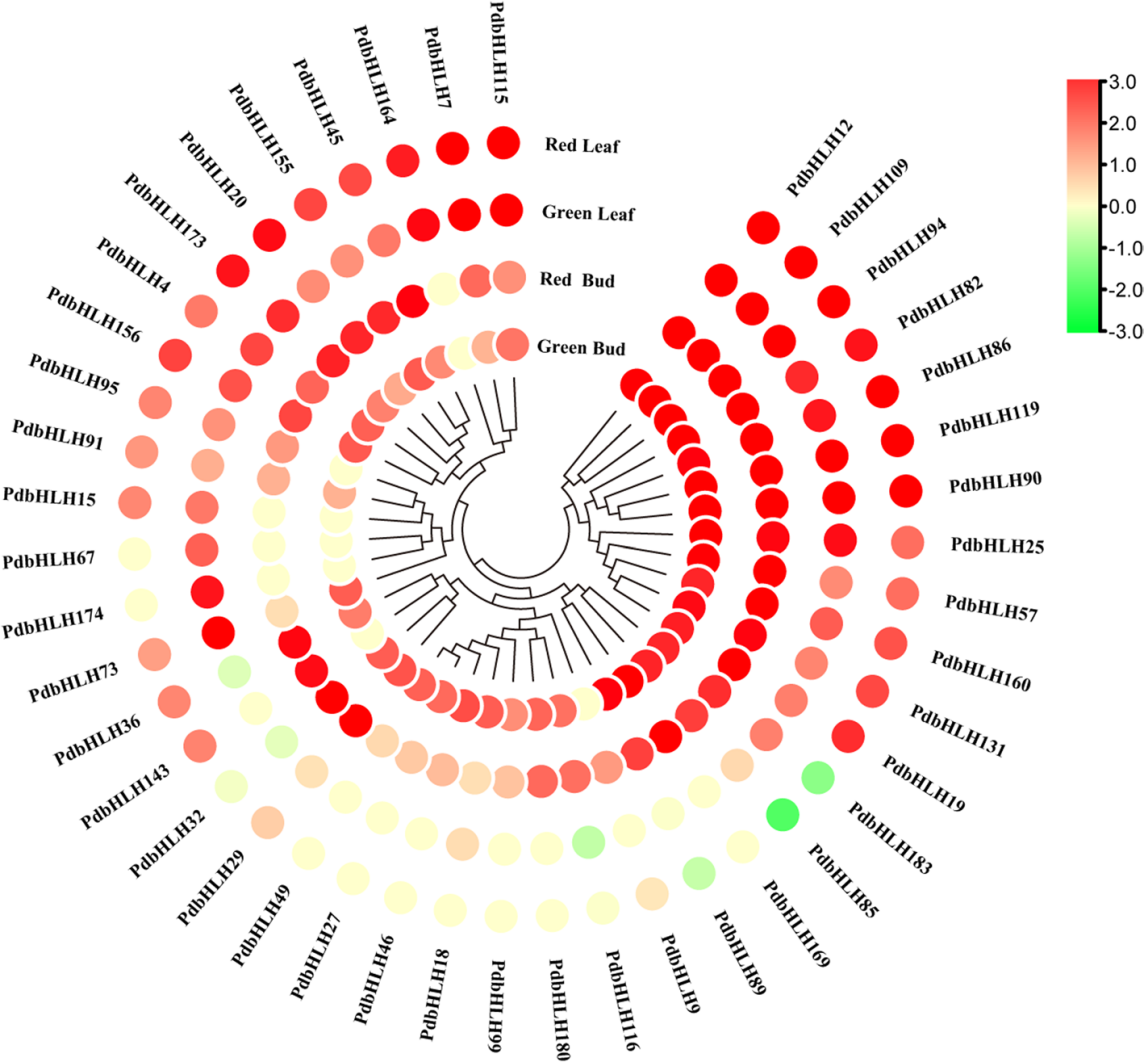
Expression profle of *PdbHLH* genes in the leaves and buds of the QHP and L2025. QHP-L, QHP leaf; L2025-L, L2025 leaf; QHP-B, QHP bud; L2025-B, L2025 bud

To further verify the expression pattern of *PdbHLH* genes, the expression level of *PdbHLH* genes in the leaves and buds of QHP and L2025 was evaluated using the previous RNA-seq data. A total of 102 *PdbHLH* genes were detected based on the RNA-seq analysis in leaves and buds of QHP and L2025 (Fig. 7, S5 and Table S9). Among them, the expression levels of 21 *PdbHLH* genes in the leaves and buds of QHP were significantly higher than that of L2025, and 2 genes (*PdbHLH143* and *PdbHLH9*) were specifically expressed in the leaves and buds of QHP, indicating that these 2 genes might be involved in the positive regulation of anthocyanin biosynthesis. *PdPdbHLH123* gene was specifically expressed in the buds of poplar, and the expression level of this gene in the buds of QHP was more than 16 times than that of L2025. Moreover, the expression level of 20 *PdbHLH* genes in buds and leaves of L2025 were higher than those in QHP (Table S9). Among them, the expression level of 3 *PdbHLH* genes (*PdbHLH183*, *PdbHLH73*, and *PdbHLH85*) in leaves of L2025 were more than 5 times than those in QHP, and 2 genes (*PdbHLH67* and *PdbHLH74*) were specifically expressed in the leaves of L2025.

Combined with the comparative transcriptome data between the green leaf poplar and colored-leaf poplar (JHP and QHP), the candidate genes associated with leaf coloration were further screened (Table S10). The expression level of 12 *PdbHLH* genes (*PdbHLH12*, *PdbHLH131*, *PdbHLH143*, *PdbHLH156*, *PdbHLH160*, *PdbHLH173*, *PdbHLH20*, *PdbHLH57*, *PdbHLH7*, *PdbHLH82*, *PdbHLH91*, and *PdbHLH95*) in leaves of JHP was more than eight times than those in L2025, and their expression in leaves of QHP was different from those in L2025, suggesting that these genes may be involved in positively regulating the anthocyanin biosynthesis. In addition, the expression level 4 *PdbHLH* genes (*PdbHLH4*, *PdbHLH1*, *PdbHLH18*, and *PdbHLH164*) in leaves of L2025 was more than 10 times than those in JHP, and its expression in leaves of L2025 was slightly higher than those in QHP, suggesting that these genes may be involved in negatively regulating the anthocyanin biosynthesis. Therefore, these 16 *PdbHLH* genes might be involved in the anthocyanin biosynthesis in leaf coloration in poplar.

### Validation of RNA-Seq-based gene expression

To validate the reliability of RNA-seq results, RT-PCR was performed on 9 genes associated with anthocyanin biosynthesis selected at random with high or low expression levels. Expression comparisons were performed in the leaves of L2025, QHP and JHP, and the expression trends in RT-PCR results were in agreement with the RNA-Seq data (Fig. S6).

### Protein interaction prediction

**Fig. 8:**
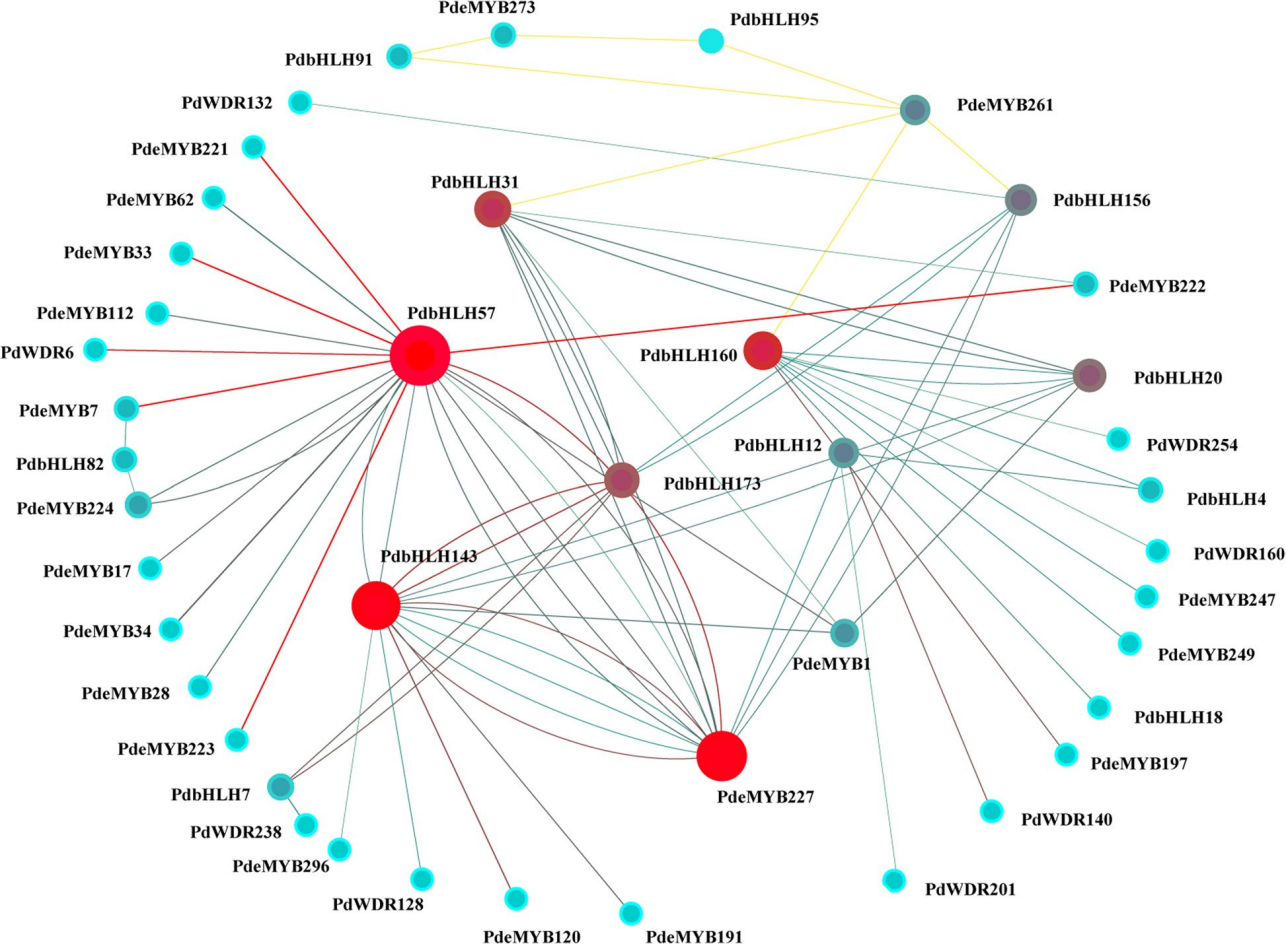
Protein interaction network analysis for the candidate PdbHLHs associated with anthocyanin biosynthesis. The online tool STRING was used to predict the network

Different bHLH proteins can form homodimers or heterodimers to bind DNA and regulate the transcription of downstream targets, which can interact with MYB and WD40 TFs to form a ternary complex (MBW) that regulates the expression of anthocyanin biosynthesis and structural genes [43, 44]. Thus, to further investigate the interaction networks of candidate PdbHLH TFs, protein interaction networks of sixteen PdbHLH proteins associated with anthocyanin biosynthesis were conducted. In our study, 16 candidate *PdbHLH* genes associated with leaf coloration were used to perform protein interaction networks, and 11 of them interacted with more than one protein (Fig. 8 and Table S11). In particular, two PdbHLH proteins (PdbHLH131, PdbHLH156) can interact with three kinds of PdMYBs, PdWDs and PdbHLHs (Fig. 8). PdbHLH20, PdbHLH173 and PdbHLH57 can interact with two kinds of PdMYBs, PdWDs and PdbHLHs, and the left ones can only interact with one kind of PdMYBs, PdWDs and PdbHLHs. However, most of them interact with PdMYBs. Interestingly, PdbHLH57, corresponding to PdTT8 associated with anthocyanin biosynthesis, can interact with many kinds of PdMYBs (such as PdMYB117, PdMYB112) and PdWDR6. It is very interesting to explore the regulation of MYB-bHLH-WDR40 model in the anthocyanin biosynthesis in poplar.

## Discussion

The bHLH transcription factors comprise a large family in higher plants, and numerous studies have shown that bHLH transcription factors are involved in diverse biological processes in plant growth, development, and stress responses [45]. However, a systematic characterization of *bHLH* genes in *P. deltoids* has not been performed. In this study, the genome-wide identification and characterization of *bHLH* family genes in *P. deltoids* were carried out. A total of 185 *PdbHLH* genes have been identified and divided into 15 groups based on phylogeny, gene structure and protein motif analyses.

Moreover, the number of *bHLH* family genes from lower plants (*Volvox carteri*, *Chlamydomonas reinhardtii*) to higher plants (*Oryza sativa*, *Solanum lycopersicum*, *Vitis vinifera*, *Fragaria vesca*, *Arabidopsis thaliana*, *Brassica rapa*, *P. deltoids*, and *Malus domestica*) were further investigated. There are a large number of *bHLH* family genes in higher plants, while a little number of *bHLH* family genes in lower plants. There was/were only one or few *bHLH* family gene (s) in each lower plants [17, 46]. The above results indicated that the expansion of *bHLH* gene family occurred in higher plants. Gene duplication events, including five types of gene duplication events, namely WGD (whole-genome duplication), dispersed, tandem, proximal and singleton duplications, contribute to the expansion of protein-coding gene families in plants [47–49]. Some large transcription factor gene families, such as APETALA2/ethylene-responsive factor (AP2/ERF) and WRKY, most likely expanded through WGD/segmental duplication and tandem duplication [40–52]. Conversely, the expansion of some other transcription factor gene families, such as MADS (Minichromosome Maintenance1 Agamous Deficiens Serum response factor) box gene family and NBS-LRR (nucleotide-binding site–leucine-rich repeat) gene family, are derived from the transposed duplication [44]. In our results, above 60% of the *PdbHLH* family genes in *P. deltoids* were duplicated and retained from proximal and whole genome duplication (WGD)/segmental duplication, respectively, suggesting that the WGD/segmental and proximal duplication contributed to the expansion of *PdbHLH* family genes. Gene expansion is accompanied by subfunctionalization and neofunctionalization, as well as gene expression patterns and protein-protein interactions. *PdbHLH143* and *PdbHLH148* were derived and retained from WGD/segmental duplication events. *PdbHLH143* was highly expressed in the leaves and buds of QHP, while the expression of *PdbHLH148* was not detected in the leaves and buds of QHP, which indicated that *PdbHLH143* might be involved in the anthocyanin biosynthesis in the color-leaf poplar.

The promoter of a gene contains cis-regulatory elements that can potentially reveal gene function [53]. Our result showed that the upstream promoter of bHLH genes in *P. deltoids* contained three types of cis-elements, including light-, stress-, and hormone-responsive elements (Fig. S2). Moreover, the promoters of *PdbHLH* genes contained the bHLH binding site (G-box), MYB-recognizing element (MRE), MYB-binding site (MBS), indicating that PdbHLH TFs can interact with each other or with MYB TFs or WDR40 TFs in the regulation of the growth and development in *P. deltoids,* including the anthocyanin biosynthesis, which was confirmed by the PPI network in our study (Fig. 8).

Genome-wide identification and characterization of *bHLH* family genes in many plants revealed that members of the III (f) subfamily have been proved to be involved in anthocyanin biosynthesis [28, 29, 54]. The *FabHLH29* gene of white-flesh strawberry mutant, a member of the III (f) subfamily in bHLH TFs, has been reported to be involved in the anthocyanin biosynthesis [28]. In *Freesia hybrida*, two members of III (f) subfamily in bHLH TFs, *FhGL3L* and *FhTT8L*, contributed to the biosynthesis of flavonoids and anthocyanin [55]. Moreover, bHLH proteins of III (f) subfamily were involved in regulating the anthocyanin biosynthesis by interacting with MYB and WDR. In chrysanthemums, *CmbHLH2* gene of III (f) subfamily regulates the anthocyanin biosynthesis by interacting with CmMYB6 to generate CmbHLH2*-*CmMYB6 complex, which can bind to the promoter of *CmDFR* gene [28]. VvMYC1, the first bHLH TF described in the grapevine, can control anthocyanin and proanthocyanidin biosynthesis through interacting with MYB5a, MYB5b, MYBA1/A2 and MYBPA1 [56]. In strawberry fruits, FvbHLH9 could form a HY5–bHLH9 complex with HY5 TF, which can positively regulate the anthocyanin biosynthesis [57]. PdTT8, one of bHLH transcription factors in poplar, physically interacted with PdMYB118 could form the PdMYB118-PdTT8 complex, which can regulate wound-induced the anthocyanin biosynthesis [26]. The PdbHLH57 in our study corresponding to previous PdTT8 belonged to the III (f) subfamily, and PdbHLH57 can interact with many kinds of PdMYBs, including PdMYB117 similar with PdMYB118, which is consistent with previous results. PdbHLH57 can also interact with PdWDR6, which can form PdMYB- PdbHLH57- PdWDR6 complex to regulate the anthocyanin biosynthesis. However, the detailed functions are still needed to be explored. In addition, the expression level of *PdbHLH57* in the leaves of JHP is much higher than that in the leaves of L2025, and there is a slightly higher expression level than that in the leaves of L2025, which indicated that *PdbHLH57* might play important roles in the leaf coloration of JHP. Another two *PdbHLH* genes *PdbHLH143* and *PdbHLH173*, belonging to the III (f) subfamily, the expression level of which in colored-leaf of polar was higher than these in green-leaf of poplar, indicating that these two genes might also be involved in the anthocyanin biosynthesis in poplar. In addition, nine *PdbHLH* genes (*PdbHLH20*, *PdbHLH7*, *PdbHLH12*, *PdbHLH91*, *PdbHLH95*, *PdbHLH82*, *PdbHLH156*, *PdbHLH160*, and *PdbHLH13*) did not include into the III (f) subfamily, however, the expression level of which in colored-leaf of polar was also higher than these in green-leaf of poplar, suggesting that these genes may be used as the candidate gens to regulate the anthocyanin biosynthesis. *CpbHLH1* is a transcription factor from *Chimonanthus praecox*. Overexpression of *CpbHLH1* in *Arabidopsis* and tobacco resulted in a dramatic decrease in anthocyanin accumulation by repressing the expression of late biosynthesis genes in the flavonoid biosynthesis pathway [58]. In our study, four genes (*PdbHLH4*, *PdbHLH1*, *PdbHLH18*, and *PdbHLH164*) displayed higher expression in the leaves of L2025 than those in colored-leaf poplar (JHP and QHP), which indicated that these genes might be involved in reducing the anthocyanin accumulation.

## Conclusions

In this study, 185 *PdbHLH* genes were identified in the *Populus deltoids* genome and were classified into 15 groups based on the sequence similarity and phylogenetic relationships. Conserved domain, gene structure, and evolutionary relationships of *PdbHLH* genes were also established and analyzed. Investigation of cis-regulatory elements of *PdbHLH* genes indicated that many *PdbHLH* genes are involved in the regulation of anthocyanin biosynthesis. Comprehensive analysis revealed that 12 candidate genes, including 3 genes (*PdbHLH57*, *PdbHLH143*, and *PdbHLH173*) from the subgroup III(f) and 9 gene from other groups, were positively associated with anthocyanin biosynthesis. In addition, 4 genes (*PdbHLH4*, *PdbHLH1*, *PdbHLH18*, and *PdbHLH164*) may be involved in negatively regulating the anthocyanin biosynthesis. The above results could provide a basis for the functional characterization of *bHLH* genes, and also provide candidate genes for the future improvement of leaf colorization in *Populus deltoids*.

## Supplementary Information

**Additional file 1**: **Figure S1**. Sequence logos of PdbHLH proteins.

**Additional file 2**: **Figure S2**. Cis-element analysis of *PdbHLH* genes from upstream 2000 bp sequence to the transcription start site.

**Additional file 3**: **Figure S3**. Proportion of genes originating from different replication events.

**Additional file 4**: **Figure S4**. Gene expression pattern of 185 *PdbHLH* genes in the leaves of JHP and L2025 by RNA-seq.

**Additional file 5**: **Figure S5.** Gene expression pattern of 185 *PdbHLH* genes in the buds and leaves of QHP and L2025 by RNA-seq.

**Additional file 6**: **Figure S6.** Relative expression levels of genes associate with anthocyanin biosynthesis in the leaves of L2025, JHP and QHP. Gene expression level was normalized with *ACTIN2*. All data represent the mean of three replicates with error bars indicating SD.

**Additional file 7**: **Table S1**. Specific primers used in relative quantitative real-time RT-PCR.

**Additional file 8**: **Table S2**. Information of *PdbHLH* genes identified in *Populus deltoids.*

**Additional file 9**: **Table S3.** The distribution ratio of *PdbHLH* genes on each chromosome in *P. deltoides*.

**Additional file 10**: **Table S4.** The gene ontology (GO) analysis of *PdbHLH* genes.

**Additional file 11**: **Table S5**. Segmentally duplicated *PdbHLH* gene pairs.

**Additional file 12**: **Table S6**. The orthologous relationships of the *bHLH* genes between *Arabidopsis* and *P. deltoids*.

**Additional file 13**: **Table S7**. The orthologous relationships of the *bHLH* genes between rice and *P. deltoids*.

**Additional file 14**: **Table S8**. The differentially expressed genes in JHP and L2025.

**Additional file 15**: **Table S9**. The differentially expressed genes in QHP and L2025.

**Additional file 16**: **Table S10.** The relative expression level of common genes in the leaves of L2025, JHP and QHP.

**Additional file 17**: **Table S11**. The protein-protein interaction used in this study.

## Data Availability

All data generated or analysed during this study are included in this published article and its supplementary information files as follows: 1. Zhang, F.; Zhao, J.; Wan, X.; Luo, X.; Li, W.; Sun, L.; Chen, Q. From green to red: large-scale transcriptome comparison of a bud sport in poplar (*Populus deltoides*). *Acta Physiol. Plant*. **2016**, 38, 1-16, doi:10.1007/s11738-016-2259-7. 2. Tian, Y.; Rao S.; Li Q.; Xu M.; Wang A.; Zhang, H.; Chen, J. The coloring mechanism of a novel golden variety in *Populus deltoides* based on the RGB color mode. Forestry Research 2021, 1: 5, doi:10.48130/FR-2021-0005.
