# Supplementary figures and images for: Genome-wide identification and characterization of PdbHLH transcription factors related to anthocyanin biosynthesis in colored-leaf poplar (Populus deltoids)
Source: BMC Genomics. 2022 Mar 28;23:244. doi: 10.1186/s12864-022-08460-5 (PMC8962177; doi:10.1186/s12864-022-08460-5)

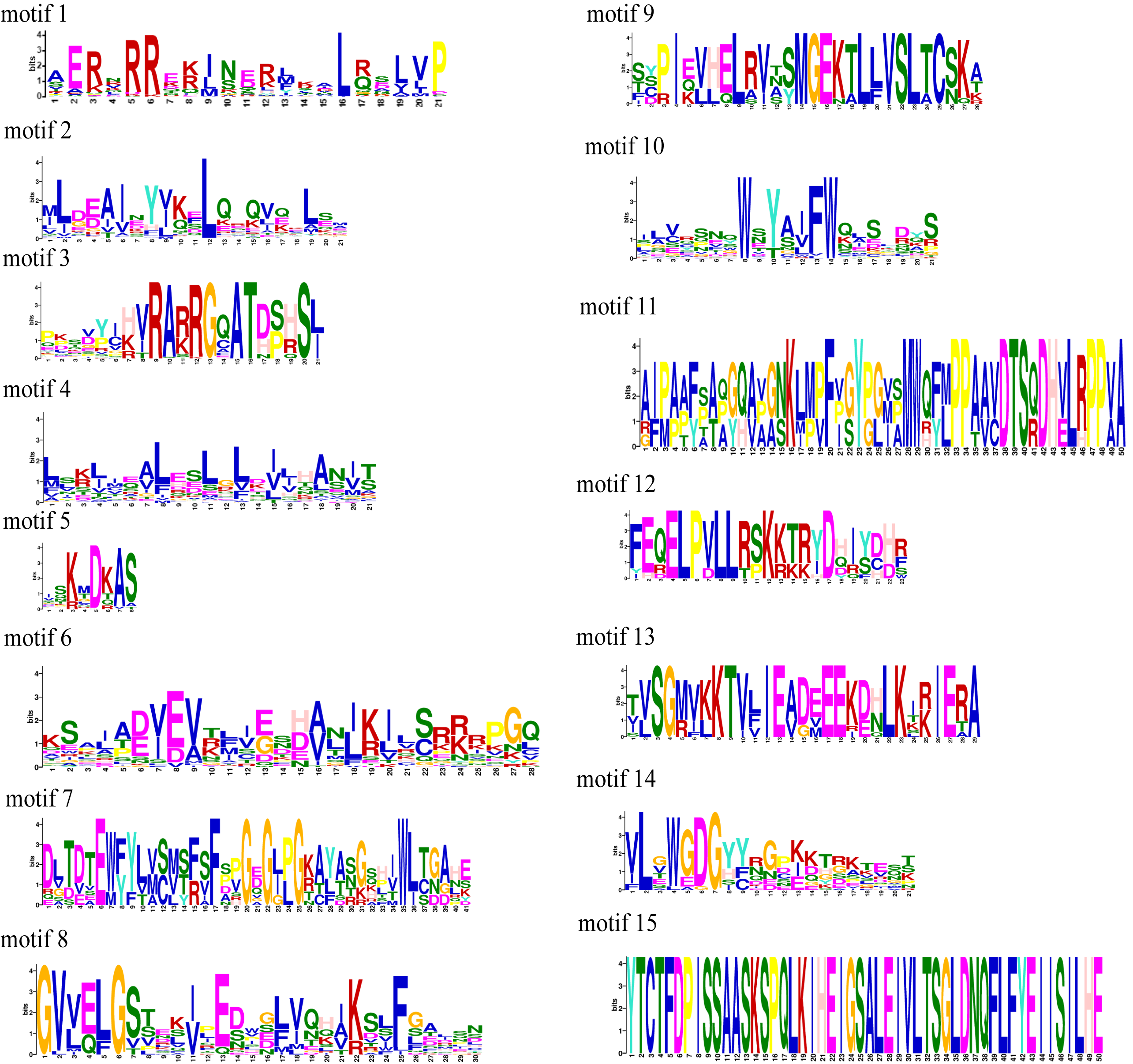

Supplement: Supplementary file 1 — Additional file 1: Figure S1. Sequence logos of PdbHLH proteins. [file 12864_2022_8460_MOESM1_ESM.tif]

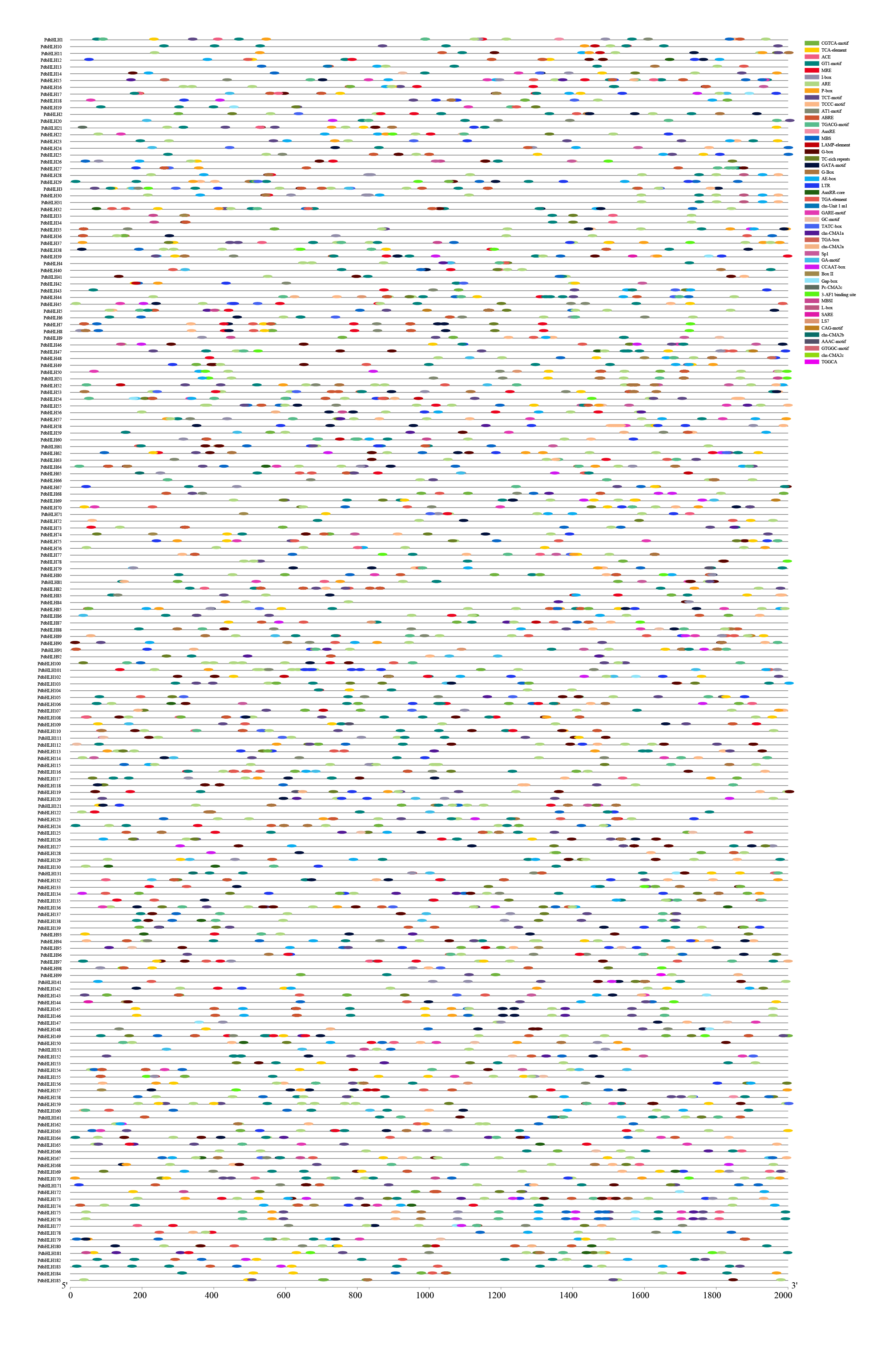

Supplement: Supplementary file 2 — Additional file 2: Figure S2. Cis-element analysis of PdbHLH genes from upstream 2000 bp sequence to the transcription start site. [file 12864_2022_8460_MOESM2_ESM.tif]

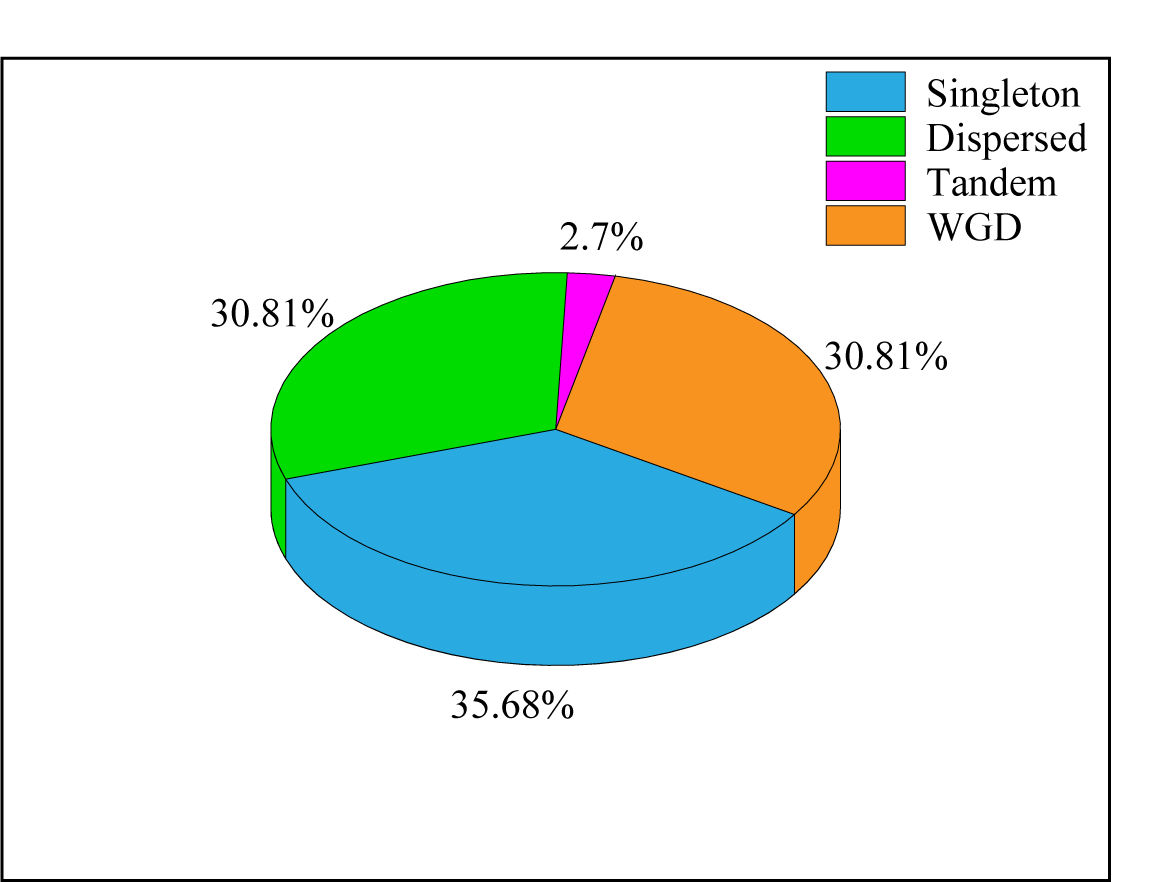

Supplement: Supplementary file 3 — Additional file 3: Figure S3. Proportion of genes originating from different replication events. [file 12864_2022_8460_MOESM3_ESM.tif]

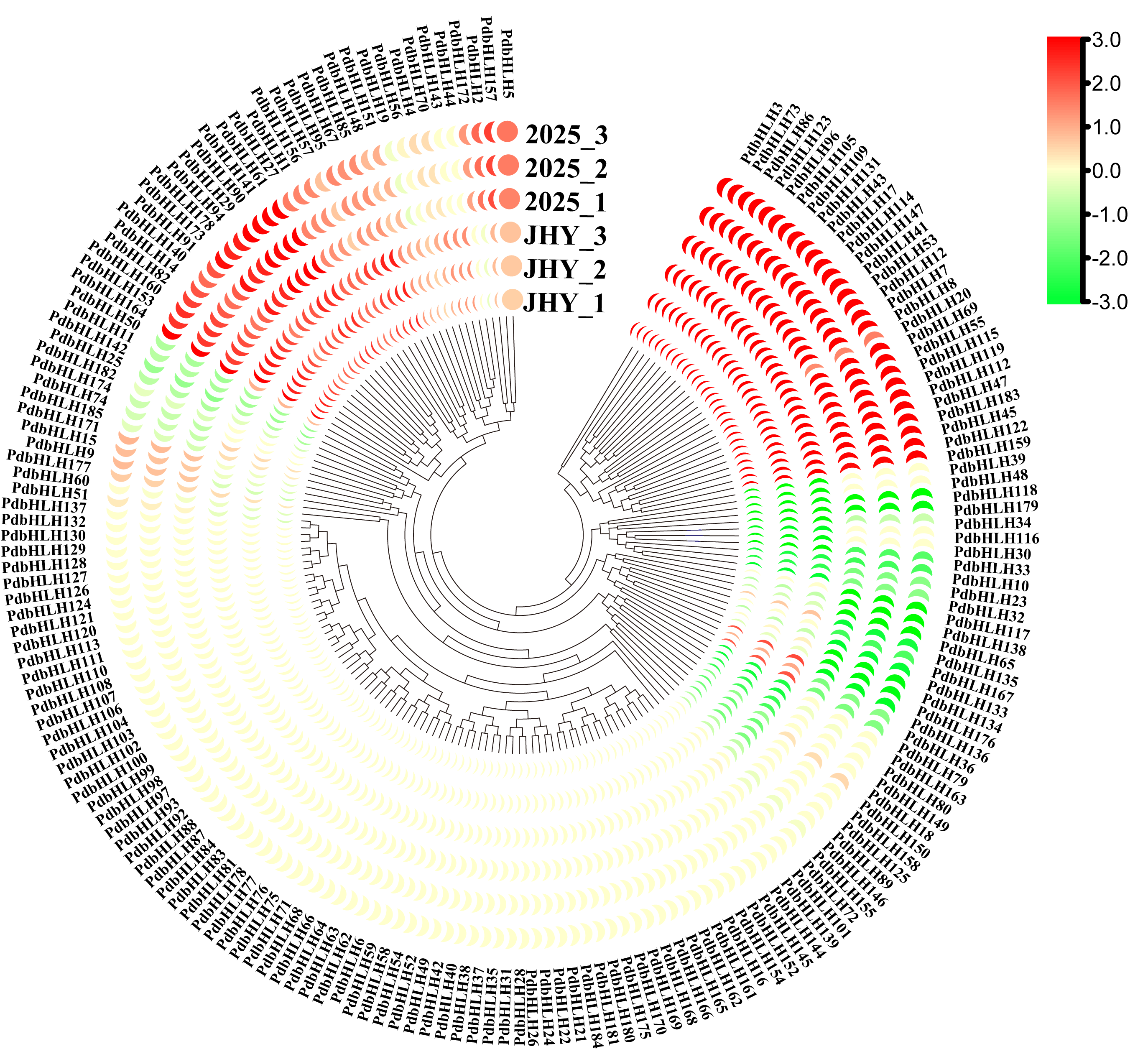

Supplement: Supplementary file 4 — Additional file 4: Figure S4. Gene expression pattern of 185 PdbHLH genes in the leaves of JHP and L2025 by RNA-seq. [file 12864_2022_8460_MOESM4_ESM.tif]

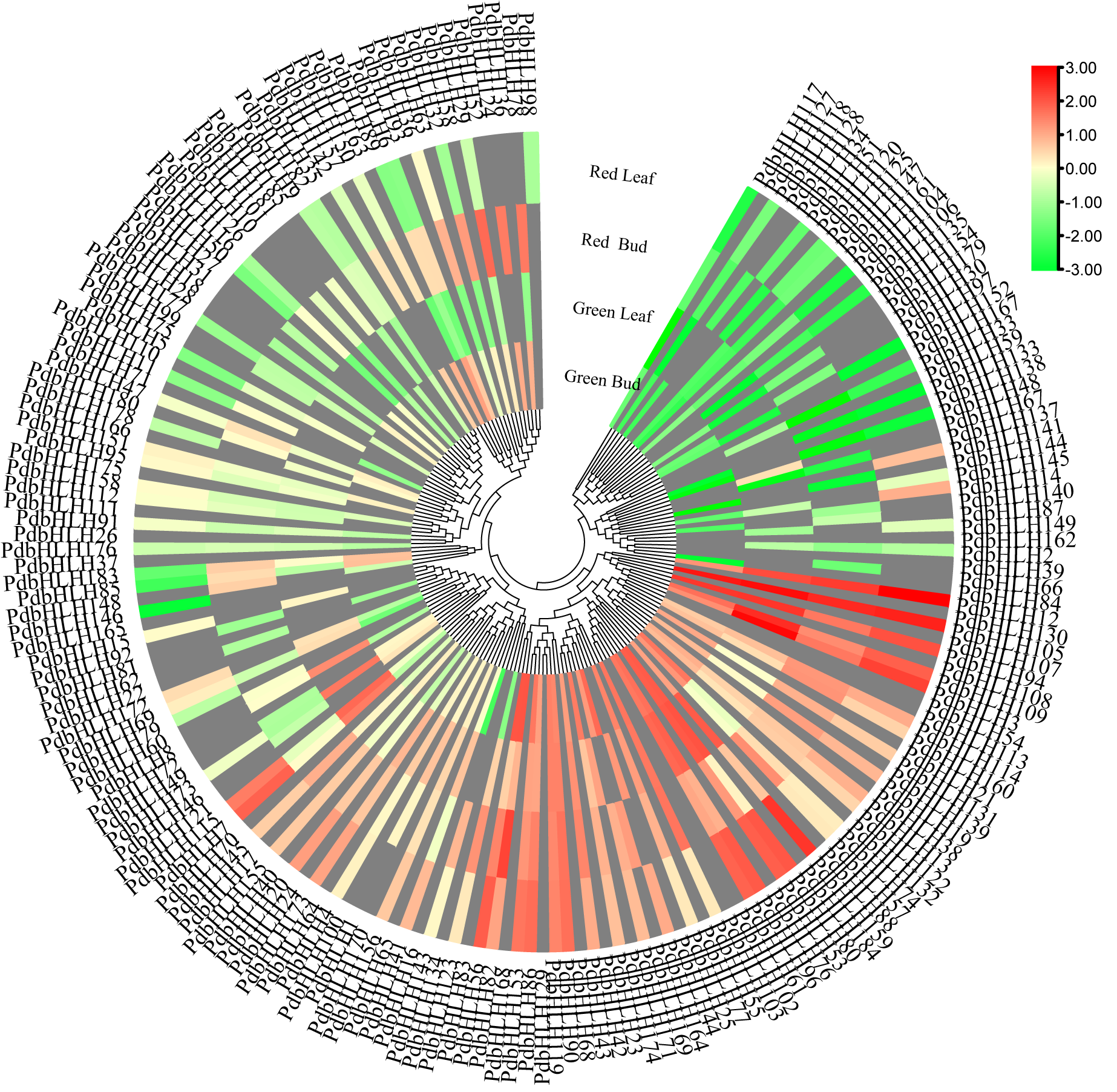

Supplement: Supplementary file 5 — Additional file 5: Figure S5. Gene expression pattern of 185 PdbHLH genes in the buds and leaves of QHP and L2025 by RNA-seq. [file 12864_2022_8460_MOESM5_ESM.tif]

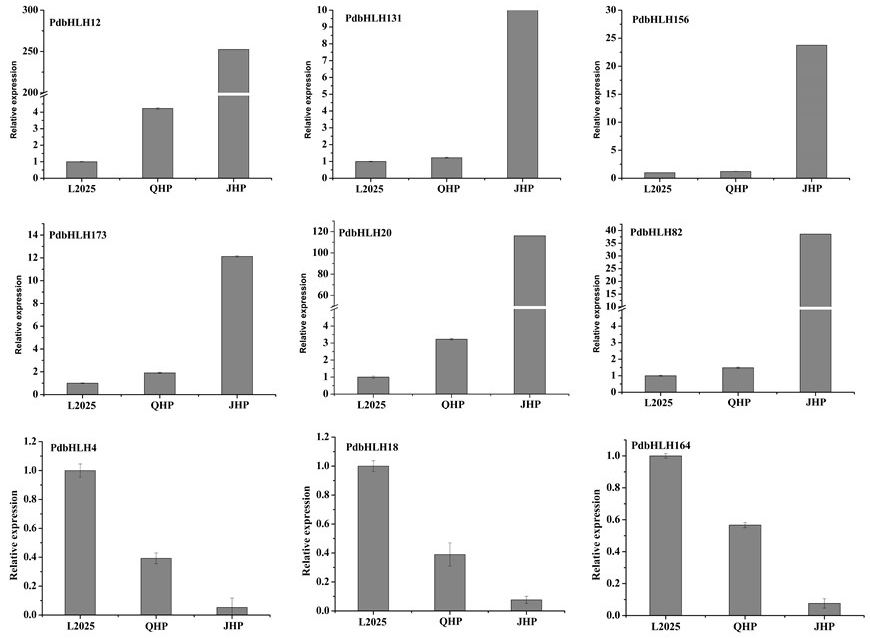

Supplement: Supplementary file 6 — Additional file 6: Figure S6. Relative expression levels of genes associate with anthocyanin biosynthesis in the leaves of L2025, JHP and QHP. Gene expression level was normalized with ACTIN2. All data represent the mean of three replicates with error bars indicating SD. [file 12864_2022_8460_MOESM6_ESM.tif]
