# Supplementary material for: Genome-wide identification and characterization of PdbHLH transcription factors related to anthocyanin biosynthesis in colored-leaf poplar (Populus deltoids)
Source: BMC Genomics. 2022 Mar 28;23:244. doi: 10.1186/s12864-022-08460-5 (PMC8962177; doi:10.1186/s12864-022-08460-5)
Supplement: Supplementary file 7 — Additional file 7: Table S1. Specific primers used in relative quantitative real-time RT-PCR. [file 12864_2022_8460_MOESM7_ESM.docx]

**Table S1**. Specific primers used in relative quantitative real-time RT-PCR.

| **Gene name** | **Accession** | **Forward primer (5’to 3’)** | **Reverse primer (5’ to 3’)** |
| --- | --- | --- | --- |
| PdbHLH12 | Podel.14G115200 | TTATGCGTGTATTGCCCAGT | TATCAAAGGAAAGTCGCCCA |
| PdbHLH131 | Podel.12G076300 | CCAACCCATCTTCCTACTCA | CCTCCTCGGAACTCAAATCT |
| PdbHLH156 | Podel.02G260000 | GCAGCTCCCATTTGGAATTC | GAGACAATGGAGCTCAAAGC |
| PdbHLH173 | Podel.15G145000 | TAACAGTGGAGGAGGTGACT | GTCCTTTAATAGCCGAGTCG |
| PdbHLH20 | Podel.02G115300 | TGGACATAAACCAGAGCCAA | TTGATACGTTGTCTGCGTCT |
| PdbHLH82 | Podel.18G114100 | TGCTGTCTACTATTCCTCCT | GCCAATATTACAGACTCATC |
| PdbHLH4 | Podel.07G000900 | CCGAAGGAAGCAAACCAACA | CCGTCAAAACCTCAACGTTG |
| PdbHLH164 | Podel.07G028600 | GACAGCAAGCGAGGTTCAAG | GTTCCCCAGATAGATTTCGG |
| PdbHLH18 | Podel.18G146400 | CATGTTCTTGAATGATGAGC | TAAGTTCCAAGAGCTTGTCA |
