# Supplementary material for: Genome-wide identification and characterization of PdbHLH transcription factors related to anthocyanin biosynthesis in colored-leaf poplar (Populus deltoids)
Source: BMC Genomics. 2022 Mar 28;23:244. doi: 10.1186/s12864-022-08460-5 (PMC8962177; doi:10.1186/s12864-022-08460-5)
Supplement: Supplementary file 8 — Additional file 8: Table S2. Information of PdbHLH genes identified in Populus deltoids. [file 12864_2022_8460_MOESM8_ESM.doc]

**Table S2** Information of *PdbHLH* genes identified in *Populus deltoids.*

| Gene ID | Gene | GenBank | Chromosome position | | Protein Properties | | | Subcellular |
| --- | --- | --- | --- | --- | --- | --- | --- | --- |
|  |  |  | start | end | length(aa) | pl | MW(KA) |  |
| PdbHLH1 | Podel.04G120100 | KAF9856569.1 | 11345474 | 11350114 | 332 | 5.29 | 36.68 | Nucleus |
| PdbHLH2 | Podel.17G104300 | KAF9830101.1 | 11466791 | 11471520 | 334 | 5.69 | 37.05 | Nucleus |
| PdbHLH3 | Podel.02G157000 | KAF9863058.1 | 11612691 | 11616821 | 479 | 8.84 | 52.24 | Nucleus |
| PdbHLH4 | Podel.07G000900 | KAF9839012.1 | 64899 | 71147 | 568 | 9.18 | 62.01 | Nucleus |
| PdbHLH5 | Podel.02G060800 | KAF9861772.1 | 4253630 | 4258003 | 530 | 6.29 | 58.03 | Nucleus |
| PdbHLH6 | Podel.14G025900 | KAF9835054.1 | 1832317 | 1835060 | 310 | 5.42 | 33.71 | Nucleus |
| PdbHLH7 | Podel.14G066900 | KAF9834237.1 | 4930358 | 4935115 | 471 | 8.58 | 51.24 | Nucleus |
| PdbHLH8 | Podel.14G059000 | - | 4317627 | 4322290 | 471 | 8.43 | 51.16 | Nucleus |
| PdbHLH9 | Podel.02G137100 | KAF9861670.1 | 10314025 | 10317147 | 329 | 5.34 | 35.03 | Nucleus |
| PdbHLH10 | Podel.13G002800 | KAF9836878.1 | 184953 | 190625 | 714 | 5.69 | 77.03 | Nucleus |
| PdbHLH11 | Podel.05G000800 | KAF9853419.1 | 44164 | 49923 | 742 | 5.89 | 79.89 | Nucleus |
| PdbHLH12 | Podel.14G115200 | KAF9834324.1 | 8471999 | 8475000 | 561 | 6.48 | 61.37 | Nucleus |
| PdbHLH13 | Podel.08G132600 | KAF9846276.1 | 8980324 | 8984421 | 329 | 5.96 | 36.41 | Nucleus |
| PdbHLH14 | Podel.05G218700 | KAF9853407.1 | 22475936 | 22476468 | 529 | 6.42 | 57.82 | Nucleus |
| PdbHLH15 | Podel.02G279700 | KAF9862491.1 | 25786105 | 25792176 | 575 | 7.27 | 62.98 | Nucleus |
| PdbHLH16 | Podel.05G153000 | KAF9855150.1 | 12904093 | 12909097 | 386 | 5.49 | 42.67 | Nucleus |
| PdbHLH17 | Podel.10G139000 | KAF9841900.1 | 13861667 | 13865820 | 320 | 5.29 | 35.41 | Nucleus |
| PdbHLH18 | Podel.18G146400 | KAF9828610.1 | 16290868 | 16292565 | 244 | 5.25 | 28.19 | Nucleus |
| PdbHLH19 | Podel.15G051800 | KAF9833036.1 | 5491278 | 5498601 | 567 | 8.44 | 61.84 | Nucleus |
| PdbHLH20 | Podel.02G115300 | KAF9861708.1 | 8473211 | 8474466 | 242 | 9.04 | 26.80 | Nucleus |
| PdbHLH21 | Podel.08G136900 | KAF9847964.1 | 9277891 | 9281263 | 358 | 5.80 | 40.05 | Nucleus |
| PdbHLH22 | Podel.16G040300 | KAF9830720.1 | 2552723 | 2555068 | 264 | 6.56 | 29.96 | Nucleus |
| PdbHLH23 | Podel.10G130000 | KAF9840860.1 | 13268666 | 13271759 | 358 | 5.92 | 39.85 | Nucleus |
| PdbHLH24 | Podel.05G220300 | KAF9862371.1 | 22627293 | 22637329 | 679 | 5.51 | 76.36 | Nucleus |
| PdbHLH25 | Podel.02G059600 | KAF9852080.1 | 4139687 | 4145880 | 695 | 4.82 | 77.90 | Nucleus |
| PdbHLH26 | Podel.06G162000 | KAF9828609.1 | 14261546 | 14265300 | 599 | 7.31 | 67.81 | Nucleus |
| PdbHLH27 | Podel.18G146500 | - | 16295274 | 16297325 | 244 | 5.35 | 28.06 | Nucleus |
| PdbHLH28 | Podel.T254000 | - | 11860 | 13762 | 236 | 8.23 | 27.42 | Nucleus |
| PdbHLH29 | Podel.10G189300 | KAF9842075.1 | 17249636 | 17250932 | 259 | 8.12 | 28.58 | Nucleus |
| PdbHLH30 | Podel.06G079900 | KAF9852251.1 | 6057405 | 6059553 | 240 | 7.64 | 27.35 | Nucleus |
| PdbHLH31 | Podel.06G080400 | - | 6132619 | 6134768 | 240 | 7.64 | 27.35 | Nucleus |
| PdbHLH32 | Podel.08G085900 | KAF9847159.1 | 5689181 | 5690790 | 259 | 9.17 | 28.55 | Nucleus |
| PdbHLH33 | Podel.06G080800 | KAF9851853.1 | 6204743 | 6209120 | 227 | 7.63 | 25.57 | Nucleus |
| PdbHLH34 | Podel.T041100 | - | 13991 | 18320 | 227 | 7.63 | 25.57 | Nucleus |
| PdbHLH35 | Podel.03G137000 | - | 14977621 | 14982773 | 634 | 5.83 | 70.35 | Nucleus |
| PdbHLH36 | Podel.13G126900 | KAF9836224.1 | 13562613 | 13564481 | 240 | 6.54 | 26.22 | Nucleus |
| PdbHLH37 | Podel.06G211200 | - | 21729394 | 21731471 | 477 | 6.87 | 53.71 | Nucleus |
| PdbHLH38 | Podel.06G037500 | KAF9853306.1 | 2643223 | 2644796 | 250 | 7.02 | 28.34 | Nucleus |
| PdbHLH39 | Podel.06G037300 | KAF9853622.1 | 2627410 | 2628915 | 251 | 7.04 | 28.32 | Nucleus |
| PdbHLH40 | Podel.15G136200 | KAF9832168.1 | 14408111 | 14409556 | 246 | 8.67 | 27.87 | Nucleus |
| PdbHLH41 | Podel.18G147000 | KAF9828328.1 | 16341494 | 16346162 | 249 | 5.16 | 28.13 | Nucleus |
| PdbHLH42 | Podel.16G072800 | KAF9831742.1 | 5248412 | 5250628 | 468 | 5.33 | 52.20 | Nucleus |
| PdbHLH43 | Podel.02G191900 | KAF9861877.1 | 14706859 | 14708715 | 618 | 6.37 | 68.29 | Nucleus |
| PdbHLH44 | Podel.14G101600 | KAF9835188.1 | 7355636 | 7359666 | 618 | 6.12 | 68.02 | Nucleus |
| PdbHLH45 | Podel.19G093200 | KAF9826021.1 | 12070854 | 12073019 | 267 | 8.25 | 29.40 | Nucleus |
| PdbHLH46 | Podel.12G034000 | KAF9837675.1 | 2738226 | 2741668 | 347 | 5.02 | 38.17 | Nucleus |
| PdbHLH47 | Podel.06G037200 | KAF9855722.1 | 2616727 | 2618985 | 240 | 8.55 | 27.19 | Nucleus |
| PdbHLH48 | Podel.01G086900 | KAF9868930.1 | 6851159 | 6853022 | 471 | 7.76 | 52.64 | Nucleus |
| PdbHLH49 | Podel.15G023000 | KAF9833227.1 | 1859036 | 1861666 | 345 | 5.03 | 37.91 | Nucleus |
| PdbHLH50 | Podel.13G112200 | KAF9837506.1 | 12162112 | 12173748 | 260 | 8.32 | 28.33 | Nucleus |
| PdbHLH51 | Podel.13G115300 | - | 12476915 | 12488129 | 260 | 8.32 | 28.33 | Nucleus |
| PdbHLH52 | Podel.03G098000 | KAF9859314.1 | 11709430 | 11713680 | 658 | 5.49 | 72.15 | Nucleus |
| PdbHLH53 | Podel.01G153100 | KAF9864641.1 | 12485744 | 12486076 | 562 | 5.26 | 61.57 | Nucleus |
| PdbHLH54 | Podel.01G323900 | - | 33161631 | 33163792 | 296 | 5.35 | 32.88 | Nucleus |
| PdbHLH55 | Podel.02G044400 | KAF9861332.1 | 3025044 | 3026555 | 503 | 6.82 | 54.99 | Nucleus |
| PdbHLH56 | Podel.02G196200 | KAF9863609.1 | 15307766 | 15309681 | 491 | 5.39 | 54.67 | Nucleus |
| PdbHLH57 | Podel.01G109500 | KAF9864213.1 | 8831527 | 8836243 | 628 | 6.26 | 69.74 | Nucleus |
| PdbHLH58 | Podel.02G118700 | KAF9863867.1 | 8851468 | 8852786 | 333 | 4.62 | 37.96 | Nucleus |
| PdbHLH59 | Podel.09G141400 | KAF9844426.1 | 11269468 | 11272470 | 587 | 5.07 | 66.29 | Nucleus |
| PdbHLH60 | Podel.16G054500 | KAF9830923.1 | 3610465 | 3613475 | 353 | 9.22 | 39.15 | Nucleus |
| PdbHLH61 | Podel.08G190100 | KAF9846755.1 | 13489760 | 13492993 | 218 | 5.64 | 24.53 | Nucleus |
| PdbHLH62 | Podel.07G012600 | - | 1023599 | 1024474 | 229 | 9.46 | 25.42 | Nucleus |
| PdbHLH63 | Podel.07G011500 | KAF9848462.1 | 949254 | 952050 | 329 | 9.16 | 36.51 | Nucleus |
| PdbHLH64 | Podel.06G149100 | KAF9852355.1 | 12562950 | 12568540 | 511 | 4.86 | 54.95 | Nucleus |
| PdbHLH65 | Podel.01G303700 | KAF9866440.1 | 31185792 | 31188123 | 330 | 8.53 | 36.96 | Nucleus |
| PdbHLH66 | Podel.13G026300 | KAF9836378.1 | 1623261 | 1625633 | 345 | 5.97 | 39.13 | Nucleus |
| PdbHLH67 | Podel.01G333900 | KAF9868192.1 | 34497189 | 34499431 | 409 | 4.98 | 46.16 | Nucleus |
| PdbHLH68 | Podel.09G083700 | KAF9844011.1 | 7808154 | 7810203 | 337 | 7.20 | 37.84 | Nucleus |
| PdbHLH69 | Podel.01G018700 | KAF9864132.1 | 1328419 | 1331274 | 414 | 6.68 | 45.99 | Nucleus |
| PdbHLH70 | Podel.03G225600 | KAF9860753.1 | 21678215 | 21681799 | 449 | 7.05 | 49.96 | Nucleus |
| PdbHLH71 | Podel.04G046300 | - | 3799857 | 3800811 | 184 | 6.45 | 20.92 | Nucleus |
| PdbHLH72 | Podel.06G080500 | KAF9852510.1 | 6166821 | 6167713 | 129 | 4.70 | 14.95 | Nucleus |
| PdbHLH73 | Podel.18G146600 | KAF9828775.1 | 16308866 | 16311032 | 247 | 5.94 | 28.97 | Nucleus |
| PdbHLH74 | Podel.14G107000 | KAF9834363.1 | 7847157 | 7849194 | 493 | 5.91 | 54.64 | Nucleus |
| PdbHLH75 | Podel.03G224200 | KAF9860753.1 | 21562999 | 21566915 | 545 | 7.99 | 61.10 | Nucleus |
| PdbHLH76 | Podel.14G026000 | KAF9835864.1 | 1838770 | 1841089 | 294 | 8.68 | 32.54 | Nucleus |
| PdbHLH77 | Podel.02G111600 | KAF9861937.1 | 8174052 | 8176157 | 326 | 5.73 | 36.72 | Nucleus |
| PdbHLH78 | Podel.05G043100 | KAF9853874.1 | 2964334 | 2966232 | 345 | 6.01 | 39.12 | Nucleus |
| PdbHLH79 | Podel.13G076300 | KAF9829740.1 | 6245191 | 6247704 | 407 | 5.13 | 46.42 | Nucleus |
| PdbHLH80 | Podel.03G099000 | KAF9859452.1 | 11827507 | 11830101 | 315 | 9.17 | 35.27 | Nucleus |
| PdbHLH81 | Podel.08G219500 | KAF9847639.1 | 15867594 | 15870589 | 488 | 8.20 | 54.52 | Nucleus |
| PdbHLH82 | Podel.18G114100 | KAF9828147.1 | 13751612 | 13755267 | 456 | 5.80 | 47.88 | Nucleus |
| PdbHLH83 | Podel.06G195600 | KAF9850225.1 | 20086542 | 20089973 | 452 | 6.14 | 47.58 | Nucleus |
| PdbHLH84 | Podel.12G139400 | KAF9838432.1 | 14536241 | 14539007 | 240 | 8.91 | 27.44 | Nucleus |
| PdbHLH85 | Podel.05G233600 | KAF9854819.1 | 23676943 | 23678583 | 504 | 6.13 | 55.42 | Nucleus |
| PdbHLH86 | Podel.14G159100 | KAF9834021.1 | 11704747 | 11712379 | 342 | 8.86 | 36.40 | Nucleus |
| PdbHLH87 | Podel.05G106200 | KAF9854804.1 | 7606321 | 7609256 | 317 | 5.66 | 35.41 | Nucleus |
| PdbHLH88 | Podel.15G136300 | KAF9833070.1 | 14419932 | 14422948 | 247 | 5.93 | 27.90 | Nucleus |
| PdbHLH89 | Podel.16G038200 | KAF9830603.1 | 2327709 | 2330069 | 241 | 8.54 | 27.18 | Nucleus |
| PdbHLH90 | Podel.10G067000 | KAF9842906.1 | 8489946 | 8493229 | 221 | 5.40 | 24.58 | Nucleus |
| PdbHLH91 | Podel.19G083100 | KAF9827023.1 | 11266728 | 11271725 | 258 | 6.05 | 28.28 | Nucleus |
| PdbHLH92 | Podel.05G077100 | KAF9855082.1 | 5227401 | 5229491 | 315 | 8.24 | 34.96 | Nucleus |
| PdbHLH93 | Podel.01G434300 | KAF9865018.1 | 46765360 | 46767927 | 252 | 8.28 | 27.93 | Nucleus |
| PdbHLH94 | Podel.15G111800 | KAF9832689.1 | 12662168 | 12666522 | 558 | 5.88 | 61.17 | Nucleus |
| PdbHLH95 | Podel.06G060000 | KAF9852765.1 | 4459781 | 4462136 | 322 | 8.90 | 35.72 | Nucleus |
| PdbHLH96 | Podel.12G113200 | KAF9839179.1 | 12649032 | 12653290 | 546 | 5.96 | 60.39 | Nucleus |
| PdbHLH97 | Podel.05G126500 | KAF9853952.1 | 9477683 | 9480237 | 318 | 5.77 | 35.19 | Nucleus |
| PdbHLH98 | Podel.01G151900 | KAF9865608.1 | 12341670 | 12344456 | 325 | 9.22 | 36.99 | Nucleus |
| PdbHLH99 | Podel.07G104100 | KAF9849165.1 | 12631175 | 12634247 | 308 | 6.68 | 34.42 | Nucleus |
| PdbHLH100 | Podel.09G003600 | KAF9844626.1 | 692194 | 693861 | 315 | 4.98 | 35.39 | Nucleus |
| PdbHLH101 | Podel.04G171900 | KAF9856038.1 | 18439583 | 18443471 | 330 | 5.76 | 36.54 | Nucleus |
| PdbHLH102 | Podel.T122400 | KAF9841900.1 | 37584 | 40900 | 400 | 6.92 | 44.92 | Nucleus |
| PdbHLH103 | Podel.08G232700 | KAF9845791.1 | 17207937 | 17209478 | 206 | 6.65 | 23.08 | Nucleus |
| PdbHLH104 | Podel.02G199200 | KAF9861123.1 | 15691385 | 15694038 | 391 | 6.62 | 44.42 | Nucleus |
| PdbHLH105 | Podel.11G130100 | KAF9840474.1 | 15654010 | 15657112 | 249 | 7.70 | 27.51 | Nucleus |
| PdbHLH106 | Podel.14G016700 | KAF9834417.1 | 1249675 | 1251773 | 362 | 4.77 | 40.23 | Nucleus |
| PdbHLH107 | Podel.01G119800 | KAF9865451.1 | 9648933 | 9650451 | 241 | 8.97 | 27.71 | Nucleus |
| PdbHLH108 | Podel.08G239300 | - | 18623122 | 18624668 | 324 | 4.92 | 35.80 | Nucleus |
| PdbHLH109 | Podel.11G030000 | KAF9839799.1 | 2649468 | 2651859 | 243 | 5.69 | 27.01 | Nucleus |
| PdbHLH110 | Podel.01G313500 | KAF9867367.1 | 32296043 | 32297971 | 366 | 4.89 | 39.87 | Nucleus |
| PdbHLH111 | Podel.09G091700 | KAF9844955.1 | 8404184 | 8405825 | 361 | 4.84 | 38.97 | Nucleus |
| PdbHLH112 | Podel.09G134600 | KAF9843962.1 | 10892691 | 10895494 | 323 | 6.38 | 35.68 | Nucleus |
| PdbHLH113 | Podel.04G030300 | - | 2406467 | 2407585 | 209 | 6.65 | 23.62 | Nucleus |
| PdbHLH114 | Podel.10G032900 | KAF9840872.1 | 5536567 | 5539500 | 241 | 6.37 | 27.08 | Nucleus |
| PdbHLH115 | Podel.04G032300 | KAF9856225.1 | 2528940 | 2531370 | 243 | 5.39 | 27.17 | Nucleus |
| PdbHLH116 | Podel.08G220500 | - | 15981232 | 15984223 | 269 | 6.60 | 30.35 | Nucleus |
| PdbHLH117 | Podel.06G111600 | KAF9851578.1 | 8993215 | 8993905 | 165 | 6.58 | 18.33 | Nucleus |
| PdbHLH118 | Podel.16G126400 | KAF9831816.1 | 12775987 | 12776722 | 163 | 6.90 | 18.16 | Nucleus |
| PdbHLH119 | Podel.02G255100 | KAF9861047.1 | 23668052 | 23676050 | 345 | 7.10 | 36.91 | Nucleus |
| PdbHLH120 | Podel.06G080700 | KAF9851117.1 | 6174587 | 6176351 | 244 | 6.41 | 27.96 | Nucleus |
| PdbHLH121 | Podel.02G130400 | KAF9863665.1 | 9837845 | 9840052 | 364 | 4.66 | 40.50 | Nucleus |
| PdbHLH122 | Podel.15G071000 | KAF9832205.1 | 9135635 | 9139013 | 274 | 5.82 | 30.16 | Nucleus |
| PdbHLH123 | Podel.09G066400 | KAF9844661.1 | 6569094 | 6572483 | 422 | 7.12 | 46.37 | Nucleus |
| PdbHLH124 | Podel.14G109600 | KAF9835168.1 | 8019298 | 8022071 | 390 | 5.33 | 43.91 | Nucleus |
| PdbHLH125 | Podel.11G049600 | KAF9839795.1 | 4414845 | 4416199 | 183 | 5.84 | 20.53 | Nucleus |
| PdbHLH126 | Podel.04G096200 | KAF9857717.1 | 8678229 | 8679946 | 334 | 5.76 | 36.77 | Nucleus |
| PdbHLH127 | Podel.17G136100 | KAF9830205.1 | 13982156 | 13983915 | 331 | 6.33 | 36.40 | Nucleus |
| PdbHLH128 | Podel.12G139500 | KAF9839104.1 | 14545336 | 14547084 | 238 | 6.51 | 27.19 | Nucleus |
| PdbHLH129 | Podel.09G022600 | - | 14545336 | 14547084 | 238 | 8.89 | 27.47 | Cytoplasm. Nucleus |
| PdbHLH130 | Podel.04G109500 | KAF9856539.1 | 10036597 | 10037909 | 178 | 9.32 | 20.17 | Nucleus |
| PdbHLH131 | Podel.12G076300 | KAF9838896.1 | 9098879 | 9102244 | 272 | 6.01 | 29.87 | Nucleus |
| PdbHLH132 | Podel.05G066300 | KAF9855643.1 | 4428018 | 4428713 | 231 | 5.82 | 26.46 | Nucleus |
| PdbHLH133 | Podel.19G105400 | KAF9826161.1 | 13110945 | 13112426 | 216 | 7.03 | 24.41 | Nucleus |
| PdbHLH134 | Podel.13G138800 | KAF9836731.1 | 14612850 | 14614057 | 222 | 5.36 | 24.91 | Chloroplast. Nucleus |
| PdbHLH135 | Podel.19G104700 | KAF9826161.1 | 13051906 | 13053383 | 236 | 8.83 | 26.75 | Nucleus |
| PdbHLH136 | Podel.17G123400 | KAF9829150.1 | 13046747 | 13049430 | 289 | 8.31 | 33.01 | Nucleus |
| PdbHLH137 | Podel.19G105300 | KAF9826371.1 | 13104395 | 13105627 | 216 | 7.03 | 24.30 | Nucleus |
| PdbHLH138 | Podel.19G104600 | - | 13045306 | 13046595 | 216 | 7.80 | 24.23 | Nucleus |
| PdbHLH139 | Podel.19G032700 | - | 3468509 | 3469599 | 188 | 5.53 | 20.65 | Nucleus |
| PdbHLH140 | Podel.13G043000 | KAF9836231.1 | 2961209 | 2965985 | 332 | 6.07 | 35.52 | Nucleus |
| PdbHLH141 | Podel.09G122300 | KAF9845342.1 | 10198462 | 10201743 | 440 | 5.58 | 48.52 | Nucleus |
| PdbHLH142 | Podel.05G058000 | KAF9855124.1 | 3943679 | 3948624 | 331 | 6.09 | 35.24 | Nucleus |
| PdbHLH143 | Podel.12G111900 | KAF9838757.1 | 12554079 | 12556985 | 359 | 7.67 | 40.30 | Nucleus |
| PdbHLH144 | Podel.07G115300 | KAF9849248.1 | 13482233 | 13482949 | 238 | 5.74 | 27.11 | Nucleus |
| PdbHLH145 | Podel.01G202700 | KAF9867429.1 | 18302903 | 18305396 | 443 | 5.97 | 48.80 | Nucleus |
| PdbHLH146 | Podel.T265900 | - | 11164 | 14094 | 443 | 5.97 | 48.80 | Nucleus |
| PdbHLH147 | Podel.16G055000 | KAF9831831.1 | 3655780 | 3660730 | 348 | 5.79 | 38.77 | Nucleus |
| PdbHLH148 | Podel.15G110700 | KAF9833431.1 | 12564092 | 12566834 | 365 | 7.78 | 41.05 | Nucleus |
| PdbHLH149 | Podel.02G275500 | KAF9861111.1 | 25499512 | 25502685 | 563 | 5.95 | 61.34 | Nucleus |
| PdbHLH150 | Podel.02G125300 | KAF9861121.1 | 9514404 | 9518084 | 399 | 5.57 | 43.55 | Nucleus |
| PdbHLH151 | Podel.05G158800 | KAF9853895.1 | 14050745 | 14054395 | 407 | 5.44 | 44.33 | Nucleus |
| PdbHLH152 | Podel.07G053000 | KAF9849494.1 | 4880411 | 4881635 | 239 | 8.89 | 26.93 | Nucleus |
| PdbHLH153 | Podel.06G059600 | KAF9852048.1 | 4413973 | 4419197 | 349 | 5.75 | 38.41 | Nucleus |
| PdbHLH154 | Podel.01G068800 | KAF9867182.1 | 5451014 | 5453196 | 315 | 5.81 | 35.34 | Nucleus |
| PdbHLH155 | Podel.14G157400 | KAF9835682.1 | 11545194 | 11548410 | 568 | 6.74 | 62.11 | Nucleus |
| PdbHLH156 | Podel.02G260000 | - | 24299169 | 24302283 | 563 | 5.46 | 61.16 | Nucleus |
| PdbHLH157 | Podel.05G135800 | KAF9854335.1 | 10265251 | 10268335 | 377 | 5.62 | 41.95 | Nucleus |
| PdbHLH158 | Podel.08G134000 | KAF9846320.1 | 9064959 | 9069767 | 570 | 5.73 | 61.20 | Nucleus |
| PdbHLH159 | Podel.04G160100 | KAF9856069.1 | 17519550 | 17522618 | 433 | 6.42 | 47.85 | Nucleus |
| PdbHLH160 | Podel.10G137500 | KAF9841124.1 | 13772958 | 13777291 | 562 | 5.63 | 60.02 | Nucleus |
| PdbHLH161 | Podel.03G159800 | - | 16768122 | 16769517 | 429 | 8.24 | 48.22 | Nucleus |
| PdbHLH162 | Podel.03G176500 | KAF9860730.1 | 17995524 | 17997103 | 291 | 7.77 | 32.88 | Nucleus |
| PdbHLH163 | Podel.01G287100 | KAF9868958.1 | 29522818 | 29525977 | 411 | 8.81 | 45.46 | Nucleus |
| PdbHLH164 | Podel.07G028600 | KAF9848663.1 | 2246876 | 2249763 | 388 | 5.77 | 43.70 | Nucleus |
| PdbHLH165 | Podel.15G049700 | KAF9833130.1 | 5165797 | 5167314 | 271 | 6.26 | 30.65 | Nucleus |
| PdbHLH166 | Podel.01G479100 | - | 51630249 | 51635594 | 326 | 5.28 | 36.42 | Nucleus |
| PdbHLH167 | Podel.08G186400 | KAF9846415.1 | 13210061 | 13212816 | 222 | 9.00 | 25.52 | Nucleus |
| PdbHLH168 | Podel.06G080000 | - | 6078451 | 6079901 | 221 | 7.02 | 25.04 | Nucleus |
| PdbHLH169 | Podel.02G176100 | KAF9861597.1 | 13248320 | 13253016 | 654 | 5.26 | 73.52 | Nucleus |
| PdbHLH170 | Podel.06G212500 | KAF9851325.1 | 21873553 | 21875185 | 426 | 5.80 | 46.72 | Nucleus |
| PdbHLH171 | Podel.03G077200 | KAF9860884.1 | 9814683 | 9816736 | 221 | 5.79 | 24.94 | Nucleus |
| PdbHLH172 | Podel.11G127100 | KAF9840153.1 | 15373963 | 15376833 | 451 | 6.13 | 49.39 | Nucleus |
| PdbHLH173 | Podel.15G145000 | KAF9833330.1 | 14914184 | 14915618 | 241 | 6.02 | 26.47 | Nucleus |
| PdbHLH174 | Podel.T132400 | KAF9865622.1 | 3283 | 6076 | 450 | 6.47 | 49.73 | Nucleus |
| PdbHLH175 | Podel.11G079300 | - | 8447026 | 8448124 | 225 | 5.31 | 25.00 | Nucleus |
| PdbHLH176 | Podel.11G080200 | KAF9839624.1 | 8531379 | 8532589 | 239 | 5.77 | 26.61 | Nucleus |
| PdbHLH177 | Podel.04G029800 | KAF9856804.1 | 2365185 | 2368341 | 483 | 5.61 | 52.97 | Nucleus |
| PdbHLH178 | Podel.11G032200 | KAF9840188.1 | 2781901 | 2785199 | 483 | 5.81 | 53.03 | Nucleus |
| PdbHLH179 | Podel.17G041900 | KAF9829099.1 | 3517173 | 3518123 | 316 | 4.90 | 34.66 | Cell membrane. Nucleus |
| PdbHLH180 | Podel.03G157300 | KAF9860217.1 | 16619955 | 16624861 | 822 | 4.99 | 90.22 | Nucleus |
| PdbHLH181 | Podel.01G089500 | KAF9865467.1 | 7050253 | 7055039 | 823 | 4.84 | 90.38 | Nucleus |
| PdbHLH182 | Podel.05G236300 | - | 23852207 | 23856813 | 583 | 5.63 | 64.32 | Nucleus |
| PdbHLH183 | Podel.05G234900 | KAF9854000.1 | 23747834 | 23752972 | 694 | 5.84 | 77.22 | Nucleus |
| PdbHLH184 | Podel.02G042900 | KAF9862041.1 | 2915421 | 2919986 | 603 | 6.59 | 67.22 | Nucleus |
| PdbHLH185 | Podel.09G016100 | KAF9867703.1 | 2640647 | 2647070 | 943 | 6.35 | 103.49 | Chloroplast. |
