# Supplementary material for: Genome-wide identification and characterization of PdbHLH transcription factors related to anthocyanin biosynthesis in colored-leaf poplar (Populus deltoids)
Source: BMC Genomics. 2022 Mar 28;23:244. doi: 10.1186/s12864-022-08460-5 (PMC8962177; doi:10.1186/s12864-022-08460-5)
Supplement: Supplementary file 9 — Additional file 9: Table S3. The distribution ratio of PdbHLH genes on each chromosome in P. deltoides. [file 12864_2022_8460_MOESM9_ESM.docx]

**Table S3** The distribution ratio of *PdebHLH* genes on each chromosome in *P. deltoides.*

| **Chr** | **Number of genes** | **Number of *PdebHLHs*** | **Percentage of *PdebHLHs* in the chromosome (%)** |
| --- | --- | --- | --- |
| 1 | 4994 | 16 | 0.32 |
| 2 | 2922 | 19 | 0.65 |
| 3 | 2466 | 9 | 0.36 |
| 4 | 2442 | 9 | 0.37 |
| 5 | 2779 | 15 | 0.54 |
| 6 | 2990 | 17 | 0.57 |
| 7 | 1611 | 7 | 0.43 |
| 8 | 2577 | 9 | 0.35 |
| 9 | 1762 | 9 | 0.51 |
| 10 | 2648 | 6 | 0.23 |
| 11 | 1677 | 7 | 0.42 |
| 12 | 1543 | 5 | 0.32 |
| 13 | 2035 | 8 | 0.39 |
| 14 | 2014 | 11 | 0.55 |
| 15 | 1503 | 9 | 0.60 |
| 16 | 1505 | 6 | 0.40 |
| 17 | 1713 | 4 | 0.23 |
| 18 | 1615 | 5 | 0.31 |
| 19 | 1346 | 7 | 0.52 |
