# Supplementary material for: Genome-wide identification and characterization of PdbHLH transcription factors related to anthocyanin biosynthesis in colored-leaf poplar (Populus deltoids)
Source: BMC Genomics. 2022 Mar 28;23:244. doi: 10.1186/s12864-022-08460-5 (PMC8962177; doi:10.1186/s12864-022-08460-5)
Supplement: Supplementary file 11 — Additional file 11: Table S5. Segmentally duplicated PdbHLH gene pairs. [file 12864_2022_8460_MOESM11_ESM.docx]

| Chr_1 | Seq_1 | Gene_1 | Start | End | Chr_1 | Seq_2 | Gene_2 | Start | End | Ka | Ks | Ka_Ks |
| --- | --- | --- | --- | --- | --- | --- | --- | --- | --- | --- | --- | --- |
| PdChr17 | Podel.17G104300.1 | PdbHLH2 | 11466791 | 11471520 | PdChr04 | Podel.04G120100.1 | PdbHLH1 | 11345474 | 11350114 | 0.0804 | 0.2266 | 0.3547 |
| PdChr14 | Podel.14G025900.1 | PdbHLH6 | 1832317 | 1835060 | PdChr02 | Podel.02G137100.1 | PdbHLH9 | 10314025 | 10317147 | 0.0716 | 0.2443 | 0.2929 |
| PdChr14 | Podel.14G101600.1 | PdbHLH44 | 7355636 | 7359666 | PdChr02 | Podel.02G191900.1 | PdbHLH43 | 14706859 | 14708715 | 0.0565 | 0.2598 | 0.2175 |
| PdChr12 | Podel.12G034000.1 | PdbHLH46 | 2738226 | 2741668 | PdChr15 | Podel.15G023000.1 | PdbHLH49 | 1859036 | 1861666 | 0.0376 | 0.2348 | 0.1601 |
| PdChr01 | Podel.01G086900.1 | PdbHLH48 | 6851159 | 6853022 | PdChr14 | Podel.14G107000.1 | PdbHLH74 | 7847157 | 7849194 | 0.3701 | 2.0691 | 0.1789 |
| PdChr01 | Podel.01G323900.1 | PdbHLH54 | 33161631 | 33163792 | PdChr14 | Podel.14G026000.1 | PdbHLH76 | 1838770 | 1841089 | 0.4107 | 1.0795 | 0.3805 |
| PdChr14 | Podel.14G107000.1 | PdbHLH74 | 7847157 | 7849194 | PdChr02 | Podel.02G196200.1 | PdbHLH56 | 15307766 | 15309681 | 0.0853 | 0.3074 | 0.2775 |
| PdChr02 | Podel.02G111600.1 | PdbHLH77 | 8174052 | 8176157 | PdChr07 | Podel.07G104100.1 | PdbHLH99 | 12631958 | 12634247 | 0.3820 | 1.5574 | 0.2453 |
| PdChr12 | Podel.12G139400.1 | PdbHLH84 | 14536241 | 14539007 | PdChr15 | Podel.15G136200.1 | PdbHLH40 | 14408111 | 14409556 | 0.1412 | 0.2209 | 0.6393 |
| PdChr14 | Podel.14G159100.1 | PdbHLH86 | 11704747 | 11712379 | PdChr02 | Podel.02G255100.1 | PdbHLH119 | 23668052 | 23676050 | 0.0746 | 0.2959 | 0.2521 |
| PdChr12 | Podel.12G113200.1 | PdbHLH96 | 12649032 | 12653290 | PdChr15 | Podel.15G111800.1 | PdbHLH94 | 12662168 | 12666522 | 0.0329 | 0.2398 | 0.1372 |
| PdChr14 | Podel.14G016700.1 | PdbHLH106 | 1249675 | 1251773 | PdChr02 | Podel.02G130400.1 | PdbHLH121 | 9837845 | 9840052 | 0.0892 | 0.2698 | 0.3306 |
| PdChr11 | Podel.11G030000.1 | PdbHLH109 | 2649468 | 2651859 | PdChr04 | Podel.04G032300.1 | PdbHLH115 | 2528940 | 2531370 | 0.0695 | 0.2051 | 0.3388 |
| PdChr01 | Podel.01G313500.1 | PdbHLH110 | 32296043 | 32297971 | PdChr14 | Podel.14G016700.1 | PdbHLH106 | 1249675 | 1251773 | 0.4312 | 2.3847 | 0.1808 |
| PdChr02 | Podel.02G130400.1 | PdbHLH121 | 9837845 | 9840052 | PdChr09 | Podel.09G091700.1 | PdbHLH111 | 8404184 | 8405825 | 0.3961 | 2.4881 | 0.1592 |
| PdChr11 | Podel.11G049600.1 | PdbHLH125 | 4414845 | 4416199 | PdChr04 | Podel.04G046300.1 | PdbHLH71 | 3799857 | 3800811 | 0.0949 | 0.2154 | 0.4405 |
| PdChr17 | Podel.17G136100.1 | PdbHLH127 | 13982156 | 13983915 | PdChr04 | Podel.04G096200.1 | PdbHLH126 | 8678229 | 8679946 | 0.0706 | 0.2038 | 0.3463 |
| PdChr12 | Podel.12G076300.1 | PdbHLH131 | 9098879 | 9102244 | PdChr15 | Podel.15G071000.1 | PdbHLH122 | 9135635 | 9139000 | 0.0755 | 0.1988 | 0.3797 |
| PdChr17 | Podel.17G123400.1 | PdbHLH136 | 13046747 | 13049430 | PdChr04 | Podel.04G109500.1 | PdbHLH130 | 10036597 | 10037909 | 0.0994 | 0.2253 | 0.4413 |
| PdChr12 | Podel.12G111900.1 | PdbHLH143 | 12554079 | 12556985 | PdChr15 | Podel.15G110700.1 | PdbHLH148 | 12564092 | 12566834 | 0.1136 | 0.2077 | 0.5471 |
| PdChr14 | Podel.14G157400.1 | PdbHLH155 | 11545194 | 11548410 | PdChr02 | Podel.02G260000.1 | PdbHLH156 | 24299169 | 24302283 | 0.0733 | 0.2298 | 0.3188 |
| PdChr05 | Podel.05G135800.1 | PdbHLH157 | 10265251 | 10268335 | PdChr09 | Podel.09G122300.1 | PdbHLH141 | 10198462 | 10201743 | 0.4411 | 1.5302 | 0.2883 |
| PdChr04 | Podel.04G160100.1 | PdbHLH159 | 17519550 | 17522618 | PdChr09 | Podel.09G122300.1 | PdbHLH141 | 10198462 | 10201743 | 0.0705 | 0.2711 | 0.2600 |
| PdChr01 | Podel.01G287100.1 | PdbHLH163 | 29522818 | 29525977 | PdChr09 | Podel.09G066400.1 | PdbHLH123 | 6569094 | 6572483 | 0.0954 | 0.3015 | 0.3165 |
| PdChr15 | Podel.15G049700.1 | PdbHLH165 | 5165797 | 5167314 | PdChr04 | Podel.04G109500.1 | PdbHLH130 | 10036597 | 10037909 | 0.3373 | 1.1292 | 0.2987 |
| PdChr02 | Podel.02G042900.1 | PdbHLH184 | 2915421 | 2919986 | PdChr05 | Podel.05G234900.1 | PdbHLH183 | 23747834 | 23752972 | 0.0943 | 0.2886 | 0.3268 |
| PdChr02 | Podel.02G157000.1 | PdbHLH3 | 11612691 | 11616821 | PdChr14 | Podel.14G066900.1 | PdbHLH7 | 4930358 | 4935115 | 0.0683 | 0.3324 | 0.2055 |
| PdChr05 | Podel.05G220300.1 | PdbHLH24 | 22627293 | 22637329 | PdChr02 | Podel.02G059600.1 | PdbHLH25 | 4139687 | 4145880 | 0.0994 | 0.3206 | 0.3101 |
| PdChr14 | Podel.14G107000.1 | PdbHLH74 | 7847157 | 7849194 | PdChr01 | Podel.01G086900.1 | PdbHLH48 | 6851159 | 6853022 | 0.3701 | 2.0691 | 0.1789 |
| PdChr07 | Podel.07G104100.1 | PdbHLH99 | 12631958 | 12634247 | PdChr02 | Podel.02G111600.1 | PdbHLH77 | 8174052 | 8176157 | 0.3820 | 1.5574 | 0.2453 |
| PdChr09 | Podel.09G091700.1 | PdbHLH111 | 8404184 | 8405825 | PdChr01 | Podel.01G313500.1 | PdbHLH110 | 32296043 | 32297971 | 0.0815 | 0.2098 | 0.3882 |
| PdChr03 | Podel.03G159800.1 | PdbHLH161 | 16768122 | 16769517 | PdChr14 | Podel.14G107000.1 | PdbHLH74 | 7847157 | 7849194 | 0.3778 | 1.6938 | 0.2231 |

**Table S5** Segmentally duplicated *PdbHLH* gene pairs.
