# Supplementary material for: Genome-wide identification and characterization of PdbHLH transcription factors related to anthocyanin biosynthesis in colored-leaf poplar (Populus deltoids)
Source: BMC Genomics. 2022 Mar 28;23:244. doi: 10.1186/s12864-022-08460-5 (PMC8962177; doi:10.1186/s12864-022-08460-5)
Supplement: Supplementary file 12 — Additional file 12: Table S6. The orthologous relationships of the bHLH genes between Arabidopsis and P. deltoids. [file 12864_2022_8460_MOESM12_ESM.docx]

**Table S6** The orthologous relationships of the *bHLH* genes between *Arabidopsis* and *P. deltoids.*

| **Gene_1** | **ID_1** | **Chr_1** | **Gene_2** | **ID_2** | **Chr_2** | **E-value** |
| --- | --- | --- | --- | --- | --- | --- |
| AT5G38860.1 | AtbHLH70 | Chr5 | Podel.04G120100.1 | PdbHLH1 | Chr04 | 1.78E-56 |
| AT4G00050.1 | AtbHLH86 | Chr4 | Podel.02G157000.1 | PdbHLH3 | Chr02 | 1.1E-108 |
| AT5G67110.1 | AtbHLH90 | Chr5 | Podel.14G025900.1 | PdbHLH6 | Chr14 | 2.18E-37 |
| AT2G46970.1 | AtbHLH88 | Chr2 | Podel.14G115200.1 | PdbHLH12 | Chr14 | 5E-39 |
| AT2G20180.1 | AtbHLH92 | Chr2 | Podel.02G279700.1 | PdbHLH15 | Chr02 | 2.03E-86 |
| AT4G29930.3 | AtbHLH34 | Chr4 | Podel.18G146400.1 | PdbHLH18 | Chr18 | 3.36E-58 |
| AT2G41240.1 | AtbHLH58 | Chr2 | Podel.16G040300.1 | PdbHLH22 | Chr16 | 1.33E-45 |
| AT2G40200.1 | AtbHLH4 | Chr2 | Podel.10G189300.1 | PdbHLH29 | Chr10 | 2.65E-49 |
| AT2G40200.1 | AtbHLH4 | Chr2 | Podel.08G085900.1 | PdbHLH32 | Chr08 | 1.38E-50 |
| AT4G29930.3 | AtbHLH34 | Chr4 | Podel.08G085900.1 | PdbHLH32 | Chr08 | 4.95E-09 |
| AT2G41240.1 | AtbHLH58 | Chr2 | Podel.06G037300.1 | PdbHLH39 | Chr06 | 8.16E-44 |
| AT4G25400.1 | AtbHLH65 | Chr4 | Podel.06G037300.1 | PdbHLH39 | Chr06 | 4.95E-10 |
| AT2G46510.1 | AtbHLH24 | Chr2 | Podel.02G191900.1 | PdbHLH43 | Chr02 | 0 |
| AT1G01260.3 | AtbHLH29 | Chr1 | Podel.14G101600.1 | PdbHLH44 | Chr14 | 0 |
| AT5G53210.1 | AtbHLH48 | Chr5 | Podel.12G034000.1 | PdbHLH46 | Chr12 | 3.34E-116 |
| AT2G41130.1 | AtbHLH5 | Chr2 | Podel.06G037200.1 | PdbHLH47 | Chr06 | 1.08E-85 |
| AT5G53210.1 | AtbHLH48 | Chr5 | Podel.15G023000.1 | PdbHLH49 | Chr15 | 5.06E-114 |
| AT4G09180.1 | AtbHLH97 | Chr4 | Podel.13G115300.1 | PdbHLH51 | Chr13 | 5.06E-85 |
| AT4G17880.1 | AtbHLH27 | Chr4 | Podel.03G098000.1 | PdbHLH52 | Chr03 | 0 |
| AT4G17880.1 | AtbHLH27 | Chr4 | Podel.01G153100.1 | PdbHLH53 | Chr01 | 6.63E-169 |
| AT2G42280.1 | AtbHLH101 | Chr2 | Podel.16G054500.1 | PdbHLH60 | Chr16 | 9.95E-108 |
| AT4G00870.1 | AtbHLH23 | Chr4 | Podel.14G107000.1 | PdbHLH74 | Chr14 | 9.04E-100 |
| AT2G24260.1 | AtbHLH104 | Chr2 | Podel.18G114100.1 | PdbHLH82 | Chr18 | 1.24E-91 |
| AT2G24260.1 | AtbHLH104 | Chr2 | Podel.06G195600.1 | PdbHLH83 | Chr06 | 7.26E-92 |
| AT4G25400.1 | AtbHLH65 | Chr4 | Podel.12G139400.1 | PdbHLH84 | Chr12 | 5.3E-47 |
| AT1G05805.1 | AtbHLH99 | Chr1 | Podel.14G159100.1 | PdbHLH86 | Chr14 | 5.79E-96 |
| AT5G51790.3 | AtbHLH66 | Chr5 | Podel.15G136300.1 | PdbHLH88 | Chr15 | 2.09E-30 |
| AT2G41130.1 | AtbHLH5 | Chr2 | Podel.16G038200.1 | PdbHLH89 | Chr16 | 1.72E-86 |
| AT4G09180.1 | AtbHLH97 | Chr4 | Podel.19G083100.1 | PdbHLH91 | Chr19 | 4.08E-85 |
| AT2G42280.1 | AtbHLH101 | Chr2 | Podel.06G060000.1 | PdbHLH95 | Chr06 | 3.74E-103 |
| AT4G36060.1 | AtbHLH11 | Chr4 | Podel.05G126500.1 | PdbHLH97 | Chr05 | 6.12E-58 |
| AT2G28160.1 | AtbHLH31 | Chr2 | Podel.05G126500.1 | PdbHLH97 | Chr05 | 4.12E-08 |
| AT1G27740.1 | AtbHLH132 | Chr1 | Podel.14G016700.1 | PdbHLH106 | Chr14 | 1.26E-15 |
| AT2G14760.1 | AtbHLH135 | Chr2 | Podel.01G313500.1 | PdbHLH110 | Chr01 | 3.82E-70 |
| AT2G14760.1 | AtbHLH135 | Chr2 | Podel.09G091700.1 | PdbHLH111 | Chr09 | 6.59E-74 |
| AT4G21330.1 | AtbHLH32 | Chr4 | Podel.04G030300.1 | PdbHLH113 | Chr04 | 1.15E-49 |
| AT5G54680.1 | AtbHLH16 | Chr5 | Podel.04G032300.1 | PdbHLH115 | Chr04 | 5.68E-88 |
| AT2G43140.1 | AtbHLH98 | Chr2 | Podel.02G255100.1 | PdbHLH119 | Chr02 | 8.46E-67 |
| AT5G62610.1 | AtbHLH123 | Chr5 | Podel.15G071000.1 | PdbHLH122 | Chr15 | 1.96E-94 |
| AT2G46810.1 | AtbHLH53 | Chr2 | Podel.14G109600.1 | PdbHLH124 | Chr14 | 1.09E-86 |
| AT1G66470.1 | AtbHLH130 | Chr1 | Podel.04G096200.1 | PdbHLH126 | Chr04 | 7.78E-66 |
| AT5G62610.1 | AtbHLH123 | Chr5 | Podel.12G076300.1 | PdbHLH131 | Chr12 | 2.12E-91 |
| AT2G18300.3 | AtbHLH121 | Chr2 | Podel.09G122300.1 | PdbHLH141 | Chr09 | 1.57E-57 |
| AT5G50915.1 | AtbHLH110 | Chr5 | Podel.12G111900.1 | PdbHLH143 | Chr12 | 3.78E-60 |
| AT2G42300.1 | AtbHLH118 | Chr2 | Podel.16G055000.1 | PdbHLH147 | Chr16 | 2.59E-101 |
| AT5G50915.1 | AtbHLH110 | Chr5 | Podel.15G110700.1 | PdbHLH148 | Chr15 | 1.67E-58 |
| AT2G42300.1 | AtbHLH118 | Chr2 | Podel.06G059600.1 | PdbHLH153 | Chr06 | 5.55E-93 |
| AT2G18300.3 | AtbHLH121 | Chr2 | Podel.05G135800.1 | PdbHLH157 | Chr05 | 2.26E-62 |
| AT2G18300.3 | AtbHLH121 | Chr2 | Podel.04G160100.1 | PdbHLH159 | Chr04 | 5.35E-60 |
| AT4G36540.2 | AtbHLH120 | Chr4 | Podel.07G028600.1 | PdbHLH164 | Chr07 | 5.42E-56 |
| AT1G73830.1 | AtbHLH109 | Chr1 | Podel.15G049700.1 | PdbHLH165 | Chr15 | 1.92E-74 |
| AT4G00480.2 | AtbHLH10 | Chr4 | Podel.02G176100.1 | PdbHLH169 | Chr02 | 5.58E-80 |
| AT1G61660.1 | AtbHLH166 | Chr1 | Podel.04G029800.1 | PdbHLH177 | Chr04 | 2.7E-78 |
| AT4G21340.1 | AtbHLH164 | Chr4 | Podel.11G032200.1 | PdbHLH178 | Chr11 | 2.74E-73 |
| AT2G31280.1 | AtbHLH77 | Chr2 | Podel.05G236300.1 | PdbHLH182 | Chr05 | 3.95E-142 |
| AT2G27230.1 | AtbHLH79 | Chr2 | Podel.09G016100.1 | PdbHLH185 | Chr09 | 1.77E-103 |
