# Supplementary material for: Genome-wide identification and characterization of PdbHLH transcription factors related to anthocyanin biosynthesis in colored-leaf poplar (Populus deltoids)
Source: BMC Genomics. 2022 Mar 28;23:244. doi: 10.1186/s12864-022-08460-5 (PMC8962177; doi:10.1186/s12864-022-08460-5)
Supplement: Supplementary file 13 — Additional file 13: Table S7. The orthologous relationships of the bHLH genes between rice and P. deltoids. [file 12864_2022_8460_MOESM13_ESM.docx]

**Table S7** The orthologous relationships of the *bHLH* genes between rice and *P. deltoids.*

| Gene_1 | ID_1 | Chr_1 | Gene_2 | ID_1 | Chr_2 | E-value |
| --- | --- | --- | --- | --- | --- | --- |
| Podel.02G115300.1 | PdbHLH20 | Chr02 | Os08g37290 | OsbHLH2 | Chr8 | 2.47E-29 |
| Podel.01G109500.1 | PdbHLH57 | Chr01 | Os04g47040 | OsbHLH20 | Chr4 | 8.72E-78 |
| Podel.11G030000.1 | PdbHLH109 | Chr11 | Os07g35870 | OsbHLH27 | Chr7 | 8.52E-76 |
| Podel.02G111600.1 | PdbHLH77 | Chr02 | Os03g08930 | OsbHLH64 | Chr3 | 3.11E-80 |
| Podel.10G139000.1 | PdbHLH17 | Chr10 | Os08g38210 | OsbHLH82 | Chr8 | 3.14E-64 |
| Podel.01G018700.1 | PdbHLH69 | Chr01 | Os08g39630 | OsbHLH108 | Chr8 | 2.95E-64 |
| Podel.12G111900.1 | PdbHLH143 | Chr12 | Os08g42470 | OsbHLH118 | Chr8 | 7.69E-66 |
| Podel.02G125300.1 | PdbHLH150 | Chr02 | Os03g51910 | OsbHLH123 | Chr3 | 4.09E-61 |
| Podel.02G275500.1 | PdbHLH149 | Chr02 | Os08g41320 | OsbHLH130 | Chr8 | 1.95E-73 |
| Podel.14G016700.1 | PdbHLH106 | Chr14 | Os03g10770 | OsbHLH148 | Chr3 | 1.50E-52 |
| Podel.14G016700.1 | PdbHLH106 | Chr14 | Os07g39940 | OsbHLH149 | Chr7 | 2.01E-51 |
| Podel.04G029800.1 | PdbHLH177 | Chr04 | Os01g57580 | OsbHLH176 | Chr1 | 8.54E-56 |
| Podel.04G029800.1 | PdbHLH177 | Chr04 | Os04g53990 | OsbHLH179 | Chr4 | 1.04E-57 |
| Podel.08G132600.1 | PdbHLH13 | Chr8 | Os08g38210 | OsbHLH82 | Chr08 | 0 |
| Podel.03G137000.1 | PdbHLH35 | Chr4 | Os04g47040 | OsbHLH20 | Chr03 | 1.97E-105 |
| Podel.03G225600.1 | PdbHLH70 | Chr8 | Os08g39630 | OsbHLH108 | Chr03 | 2.18E-83 |
| Podel.09G091700.1 | PdbHLH111 | Chr3 | Os03g10770 | OsbHLH148 | Chr09 | 1.44E-59 |
| Podel.04G032300.1 | PdbHLH115 | Chr7 | Os07g35870 | OsbHLH27 | Chr04 | 2.62E-74 |
| Podel.02G130400.1 | PdbHLH121 | Chr7 | Os07g39940 | OsbHLH149 | Chr02 | 3.36E-54 |
| Podel.09G066400.1 | PdbHLH123 | Chr8 | Os08g39630 | OsbHLH108 | Chr09 | 2.15E-88 |
| Podel.15G110700.1 | PdbHLH148 | Chr8 | Os08g42470 | OsbHLH118 | Chr15 | 1.80E-60 |
| Podel.08G134000.1 | PdbHLH158 | Chr8 | Os08g38080 | OsbHLH121 | Chr08 | 3.54E-58 |
| Podel.10G137500.1 | PdbHLH160 | Chr8 | Os08g38080 | OsbHLH121 | Chr10 | 1.58E-62 |
