# Supplementary material for: Genome-wide identification and characterization of PdbHLH transcription factors related to anthocyanin biosynthesis in colored-leaf poplar (Populus deltoids)
Source: BMC Genomics. 2022 Mar 28;23:244. doi: 10.1186/s12864-022-08460-5 (PMC8962177; doi:10.1186/s12864-022-08460-5)
Supplement: Supplementary file 14 — Additional file 14: Table S8. The differentially expressed genes in JHP and L2025. [file 12864_2022_8460_MOESM14_ESM.docx]

**Table S8** The differentially expressed genes in JHP and L2025.

| **Gene Name** | **subfamily** | **Average fpkm** | | |
| --- | --- | --- | --- | --- |
|  |  | **JHP Leaves** | **L2025 Leaves** | **Fold change(JHP/L2025)** |
| PdbHLH67 | Ia | 5.682768355 | 0.173670748 | 32.723 |
| PdbHLH94 | III(a+b+c) | 4.015871689 | 0.153192525 | 26.215 |
| PdbHLH41 | III(a+b+c) | 27.71084869 | 0.153192525 | 180.889 |
| PdbHLH73 | III(a+b+c) | 19.54451802 | 0.153192525 | 127.581 |
| PdbHLH55 | III(d+e) | 10.13504236 | 0.153192525 | 66.159 |
| PdbHLH85 | III(d+e) | 5.914704689 | 0.153192525 | 38.610 |
| PdbHLH53 | III(d+e) | 41.03126236 | 0.153192525 | 267.841 |
| PdbHLH43 | III(d+e) | 23.02946036 | 0.195217415 | 117.968 |
| PdbHLH57 | IIIf | 4.523610689 | 0.153192525 | 29.529 |
| PdbHLH173 | IIIf | 2.676614022 | 0.224319081 | 11.932 |
| PdbHLH143 | IIIf | 2.294224355 | 0.153192525 | 14.976 |
| PdbHLH105 | IVc | 22.12246836 | 0.177166192 | 124.868 |
| PdbHLH109 | IVc | 17.72196369 | 0.153192525 | 115.684 |
| PdbHLH115 | IVc | 10.83567136 | 0.153192525 | 70.732 |
| PdbHLH61 | IVc | 5.290492689 | 0.153192525 | 34.535 |
| PdbHLH20 | V | 22.38498036 | 0.160788859 | 139.219 |
| PdbHLH47 | V | 7.559854355 | 0.153192525 | 49.349 |
| PdbHLH17 | Va | 15.50244569 | 0.153192525 | 101.196 |
| PdbHLH1 | Va | 3.201903689 | 0.153192525 | 20.901 |
| PdbHLH3 | VII（a+b） | 71.40508469 | 0.153192525 | 466.113 |
| PdbHLH12 | VII（a+b） | 46.04458669 | 0.153192525 | 300.567 |
| PdbHLH7 | VII（a+b） | 32.77515369 | 0.153192525 | 213.947 |
| PdbHLH8 | VII（a+b） | 28.68727102 | 0.200405081 | 143.146 |
| PdbHLH14 | VII（a+b） | 5.785481022 | 0.160433415 | 36.062 |
| PdbHLH123 | IX | 26.47690836 | 0.186855859 | 141.697 |
| PdbHLH95 | IX | 4.634177022 | 0.153192525 | 30.251 |
| PdbHLH91 | IX | 4.135021355 | 0.153192525 | 26.992 |
| PdbHLH82 | XI | 6.115532355 | 0.153192525 | 39.921 |
| PdbHLH140 | XI | 4.563580022 | 0.153192525 | 29.790 |
| PdbHLH131 | XII | 20.72462469 | 2.61244489 | 7.933 |
| PdbHLH147 | XII | 13.85745836 | 0.153192525 | 90.458 |
| PdbHLH122 | XII | 12.88223936 | 0.153192525 | 84.092 |
| PdbHLH153 | XII | 7.681920689 | 0.55188389 | 13.919 |
| PdbHLH160 | XII | 6.058840022 | 0.153192525 | 39.550 |
| PdbHLH141 | XII | 4.915052355 | 0.153192525 | 32.084 |
| PdbHLH136 | XII | 4.197683689 | 0.153192525 | 27.401 |
| PdbHLH156 | XII | 3.243248355 | 0.153192525 | 21.171 |
| PdbHLH148 | XII | 2.357669689 | 0.153192525 | 15.390 |
| PdbHLH183 | XIII | 14.14402769 | 0.153192525 | 92.328 |
| PdbHLH36 | V | 1.896720022 | 90.42131456 | 0.021 |
| PdbHLH164 | XII | 1.780585022 | 30.90971022 | 0.058 |
| PdbHLH151 | XII | 1.847883355 | 31.82536722 | 0.058 |
| PdbHLH177 | X | 1.251619022 | 16.20115289 | 0.077 |
| PdbHLH134 | VIIIa1 | 1.064653355 | 13.75627022 | 0.077 |
| PdbHLH185 | XIII | 1.224824022 | 15.81063256 | 0.077 |
| PdbHLH74 | III(d+e) | 1.332757689 | 17.09872522 | 0.078 |
| PdbHLH2 | Va | 0.879843689 | 10.97320489 | 0.080 |
| PdbHLH56 | III(d+e) | 1.429444355 | 17.14164722 | 0.083 |
| PdbHLH19 | Orphan | 1.744116689 | 20.54436156 | 0.085 |
| PdbHLH135 | VIIIa1 | 1.519068689 | 17.39249989 | 0.087 |
| PdbHLH171 | XII | 0.879351689 | 10.03176756 | 0.088 |
| PdbHLH5 | VII(a+b) | 1.542833022 | 17.43775689 | 0.088 |
| PdbHLH157 | XII | 1.697780689 | 19.12384422 | 0.089 |
| PdbHLH15 | VII(a+b) | 0.829851022 | 9.286838557 | 0.089 |
| PdbHLH101 | IVb | 1.018947355 | 11.08502756 | 0.092 |
| PdbHLH149 | XIIX | 0.224389236 | 2.439287223 | 0.092 |
| PdbHLH18 | III(a+b+c) | 0.233844792 | 2.48166489 | 0.094 |
| PdbHLH23 | V | 0.220447236 | 2.328790223 | 0.095 |
| PdbHLH10 | VII(a+b) | 0.214572459 | 2.223722223 | 0.096 |
| PdbHLH176 | XIVa | 0.825061689 | 8.475092557 | 0.097 |
| PdbHLH60 | IX | 0.824063022 | 8.461625223 | 0.097 |
| PdbHLH4 | Orphan | 1.851556022 | 81.17463422 | 0.023 |
