# Supplementary material for: Genome-wide identification and characterization of PdbHLH transcription factors related to anthocyanin biosynthesis in colored-leaf poplar (Populus deltoids)
Source: BMC Genomics. 2022 Mar 28;23:244. doi: 10.1186/s12864-022-08460-5 (PMC8962177; doi:10.1186/s12864-022-08460-5)
Supplement: Supplementary file 15 — Additional file 15: Table S9. The differentially expressed genes in QHP and L2025. [file 12864_2022_8460_MOESM15_ESM.docx]

**Table S9** The differentially expressed genes in QHP and L2025.

| **Gene Name** | **subfamily** |  | **Average** fpkm | | | |  |
| --- | --- | --- | --- | --- | --- | --- | --- |
|  |  | **Green Bud** | **Red**  Bud | **Fold change (Green/Red)** | **Green Leaf** | **Red Leaf** | **Fold change (Green/Red)** |
| PdbHLH173 | IIIf | 3.578376 | 9.554121 | 0.375 | 8.51296 | 11.1848 | 0.761 |
| PdbHLH57 | IIIf | 15.04812 | 21.11867 | 0.713 | 3.237381 | 4.357421 | 0.743 |
| PdbHLH143 | IIIf | 0 | 11.96901 | 0.000 | 0 | 3.533998 | 0.000 |
| PdbHLH90 | IVc | 17.6043 | 30.54341 | 0.576 | 16.65498 | 22.68136 | 0.734 |
| PdbHLH20 | V | 2.368853 | 9.133617 | 0.259 | 3.220673 | 12.10512 | 0.266 |
| PdbHLH29 | V | 5.727191 | 34.58439 | 0.166 | 1.343692 | 1.640619 | 0.819 |
| PdbHLH32 | V | 5.172105 | 17.67432 | 0.293 | 0.811481 | 0.914389 | 0.887 |
| PdbHLH36 | V | 3.746415 | 12.73032 | 0.294 | 0.765735 | 3.410904 | 0.224 |
| PdbHLH45 | V | 3.286497 | 13.35617 | 0.246 | 3.889595 | 6.295955 | 0.618 |
| PdbHLH12 | VII(a+b) | 15.35501 | 65.69204 | 0.234 | 32.64975 | 135.6911 | 0.241 |
| PdbHLH7 | VII(a+b) | 2.143068 | 4.598843 | 0.466 | 17.89251 | 27.21396 | 0.657 |
| PdbHLH9 | VII(a+b) | 0 | 2.791325 | 0.000 | 0 | 1.271971 | 0.000 |
| PdbHLH86 | IX | 13.39252 | 24.83377 | 0.539 | 10.69422 | 23.47892 | 0.455 |
| PdbHLH119 | IX | 18.90307 | 22.81929 | 0.828 | 16.66105 | 16.91957 | 0.985 |
| PdbHLH91 | IX | 2.183923 | 2.198659 | 0.993 | 2.253095 | 2.859965 | 0.788 |
| PdbHLH95 | IX | 0 | 2.81119 | 0.000 | 3.020744 | 3.437232 | 0.879 |
| PdbHLH82 | XI | 31.7498 | 59.28772 | 0.536 | 8.806002 | 11.12613 | 0.791 |
| PdbHLH116 | XII | 4.249157 | 4.31547 | 0.985 | 0.610448 | 0.979937 | 0.623 |
| PdbHLH131 | XII | 12.18135 | 12.97209 | 0.939 | 3.452218 | 6.396214 | 0.540 |
| PdbHLH155 | XII | 5.358984 | 8.93968 | 0.599 | 3.044283 | 6.545634 | 0.465 |
| PdbHLH156 | XII | 5.377762 | 6.557171 | 0.820 | 5.812801 | 6.760186 | 0.860 |
| PdbHLH160 | XII | 9.201289 | 19.30115 | 0.477 | 5.247799 | 5.832013 | 0.900 |
| PdbHLH19 | Orphan | 9.877651 | 16.52904 | 0.598 | 3.666388 | 8.533667 | 0.430 |
| PdbHLH109 | IVc | 23.30576 | 21.61579 | 1.078 | 48.11398 | 28.2246 | 1.705 |
| PdbHLH115 | IVc | 4.052851 | 3.083446 | 1.314 | 22.69179 | 18.81098 | 1.206 |
| PdbHLH15 | VII(a+b) | 0 | 0 |  | 3.934253 | 3.378369 | 1.165 |
| PdbHLH164 | XII | 0 | 0 |  | 12.6456 | 10.09057 | 1.253 |
| PdbHLH169 | IIIf | 24.54967 | 17.28448 | 1.420 | 0 | 0 |  |
| PdbHLH174 | X | 0 | 0 |  | 11.07013 | 0 |  |
| PdbHLH18 | III(a+b+c) | 5.168024 | 1.371794 | 3.767 | 1.406044 | 0 |  |
| PdbHLH180 | XIII | 4.802485 | 4.572477 | 1.050 | 0 | 0 |  |
| PdbHLH183 | XIII | 9.127073 | 8.496887 | 1.074 | 3.631583 | 0.393583 | 9.227 |
| PdbHLH25 | IIIf | 18.39298 | 12.75023 | 1.443 | 11.84006 | 4.375698 | 2.706 |
| PdbHLH27 | III(a+b+c) | 4.563303 | 1.730948 | 2.636 | 0 | 0 |  |
| PdbHLH4 | Orphan | 5.047023 | 4.824857 | 1.046 | 6.544695 | 3.862847 | 1.694 |
| PdbHLH46 | Ia | 6.100048 | 1.963631 | 3.107 | 0 | 0 |  |
| PdbHLH49 | Ia | 4.999972 | 1.467546 | 3.407 | 0 | 0 |  |
| PdbHLH67 | Ia | 0 | 0 |  | 5.046831 | 0 |  |
| PdbHLH73 | III(a+b+c) | 5.186752 | 1.393984 | 3.721 | 18.71916 | 2.654143 | 7.053 |
| PdbHLH85 | III(d+e) | 9.337165 | 7.141439 | 1.307 | 1.463602 | 0.2428 | 6.028 |
| PdbHLH89 | V | 13.26628 | 6.956988 | 1.907 | 0.98075 | 0.628432 | 1.561 |
| PdbHLH94 | III(a+b+c) | 43.8833 | 28.66225 | 1.531 | 43.3431 | 34.49524 | 1.256 |
| PdbHLH99 | Ia | 3.136642 | 1.839114 | 1.706 | 0 | 0 |  |
