# Supplementary material for: Genome-wide identification and characterization of PdbHLH transcription factors related to anthocyanin biosynthesis in colored-leaf poplar (Populus deltoids)
Source: BMC Genomics. 2022 Mar 28;23:244. doi: 10.1186/s12864-022-08460-5 (PMC8962177; doi:10.1186/s12864-022-08460-5)
Supplement: Supplementary file 16 — Additional file 16: Table S10. The relative expression level of common genes in the leaves of L2025, JHP and QHP. [file 12864_2022_8460_MOESM16_ESM.docx]

| Gene name | Average value | | Fold change  (QHY/L2025) | Average value | | Fold change  (JHY/L2025) |
| --- | --- | --- | --- | --- | --- | --- |
|  | L2025-leaf | QHY-leaf |  | L2025-leafA | JHY-leaf |  |
| PdbHLH12 | 32.65 | 41.49 | 1.27 | 15.82 | 46.05 | 2.91 |
| PdbHLH131 | 3.45 | 17.13 | 4.96 | 13.18 | 20.73 | 1.57 |
| PdbHLH143 | 0 | 1.07 | - | 0 | 2.30 | - |
| PdbHLH156 | 5.81 | 3.38 | 0.58 | 2.47 | 3.24 | 1.31 |
| PdbHLH160 | 5.25 | 5.20 | 0.99 | 5.16 | 6.06 | 1.17 |
| PdbHLH173 | 8.51 | 5.65 | 0.66 | 6.83 | 2.68 | 0.39 |
| PdbHLH20 | 3.22 | 20.44 | 6.35 | 2.70 | 22.39 | 8.30 |
| PdbHLH57 | 3.24 | 5.78 | 1.78 | 1.57 | 4.52 | 2.88 |
| PdbHLH7 | 17.89 | 29.26 | 1.64 | 17.38 | 32.78 | 1.89 |
| PdbHLH82 | 8.81 | 5.47 | 0.62 | 4.45 | 6.12 | 1.37 |
| PdbHLH91 | 2.25 | 1.62 | 0.72 | 3.81 | 4.14 | 1.09 |
| PdbHLH9 | 0 | 0.90 | - | 1.65 | 0.71 | 0.43 |
| PdbHLH4 | 6.54 | 3.70 | 0.57 | 1.27 | 1.85 | 1.46 |
| PdbHLH1 | 7.32 | 1.11 | 0.15 | 2.60 | 3.20 | 1.23 |
| PdbHLH18 | 1.41 | 0.56 | 0.40 | 0 | 0.21 | - |
| PdbHLH164 | 12.65 | 7.86 | 0.62 | 13.75 | 1.78 | 0.13 |

**Table S10** The relative expression level of common genes in the leaves of L2025, JHP and QHP.
