# Supplementary material for: Genome-wide identification and characterization of PdbHLH transcription factors related to anthocyanin biosynthesis in colored-leaf poplar (Populus deltoids)
Source: BMC Genomics. 2022 Mar 28;23:244. doi: 10.1186/s12864-022-08460-5 (PMC8962177; doi:10.1186/s12864-022-08460-5)
Supplement: Supplementary file 17 — Additional file 17: Table S11. The protein-protein interaction used in this study. [file 12864_2022_8460_MOESM17_ESM.docx]

| Gene | MYB | WDR40 | bHLH |
| --- | --- | --- | --- |
| PdbHLH20 | PdMYB234 | - | PdbHLH143, PdbHLH131 |
| PdbHLH131 | PdMYB173, PdMYB172, PdMYB102, PdMYB3, PdMYB1, PdMYB99, PdMYB140, PdMYB31 | PdWDR128 | PdbHLH57, PdbHLH173, PdbHLH57 |
| PdbHLH156 | PdMYB261, PdMYB79, PdMYB150 | PdWDR132 | PdbHLH173 |
| PdbHLH173 | - | PdWDR6 | PdbHLH7 |
| PdbHLH57 | PdMYB28, PdMYB154, PdMYB236, PdMYB157, PdMYB3, PdMYB79, PdMYB227, PdMYB117, PdeMYB17, PdMYB9, PdMYB49, PdMYB111, PdMYB112, PdMYB33, PdMYB62, PdMYB221 | PdWDR6 | - |
| PdbHLH82 | PdMYB9, PdMYB111 | - | - |
| PdbHLH91 | PdMYB261, PdMYB273 | - | - |
| PdbHLH95 | PdMYB261, PdMYB273 | - | - |
| PdbHLH143 | PdeMYB206 | - | - |
| PdbHLH4 | - | - | PdbHLH12, PdbHLH160 |
| PdbHLH18 | - | - | PdbHLH160 |

**Table S11** The protein-protein interaction used in this study.

Note: PdMYB234, Podel.19G036300; PdMYB261, Podel.02G087800; PdMYB79, Podel.08G107000; PdMYB150, Podel.17G090200; PdMYB173, Podel.19G047500; PdMYB172, Podel.19G042700; PdMYB102, Podel.10G169100; PdMYB3, Podel.01G090000; PdMYB1, Podel.01G005800; PdMYB99, Podel.10G142600; PdMYB140, Podel.15G081400; PdMYB31, Podel.02G220800; PdMYB222, Podel.04G013300; PdMYB117, Podel.13G059800; PdMYB273, Podel.02G004500; PdMYB28, Podel.02G193100; PdMYB154, Podel.17G135000; PdMYB236, Podel.T259500; PdMYB157, Podel.17G135300; PdMYB227, Podel.10G189200; PdMYB17, Podel.01G369200; PdMYB9, Podel.01G246300; PdMYB49, Podel.04G143500; PdMYB111, Podel.12G083500; PdMYB112, Podel.12G087800; PdMYB33, Podel.03G067500; PdMYB62, Podel.06G071900; PdMYB221, Podel.02G188300; PdMYB206, Podel.08G221200; PdMYB223, Podel.04G021100; PdWDR128, Podel.18G023600; PdWDR132, Podel.14G189000; PdWDR6, Podel.13G087100;
